# Early Detection of Glottic Cancer Through Machine Learning and Acoustic Voice Analysis: A Systematic Review

**DOI:** 10.3390/medsci14050581

**Published:** 2026-09-17

**Authors:** Himanshu Verma, Roshani Mishra, Banumathy Nagamani, Sourabha Kumar Patro, Anurag Snehi Ramavat, Jaimanti Bakshi, Padmavati Khandnor, Sudesh Rani, Trilok Chand

**Affiliations:** 1 Department of Therapeutics, National Institute for the Empowerment of Persons with Intellectual Disabilities, Secunderabad 500009, India; 2 Department of Otolaryngology, Post Graduate Institute of Medical Education and Research, Chandigarh 160012, India; 279roshanimishra@gmail.com (R.M.); sourabhlipi@gmail.com (S.K.P.); ramavatanu@gmail.com (A.S.R.); drjbakshipgi@gmail.com (J.B.); 3 Department of Computer Science and Engineering, Punjab Engineering College, Chandigarh 160012, India; padmavati@pec.edu.in (P.K.); sudeshrani@pec.edu.in (S.R.); trilokchand@pec.edu.in (T.C.)

**Keywords:** glottic cancer, machine learning, acoustic features, MFCC, deep learning

## Abstract

**Purpose**: Early detection of glottic cancer is essential for better outcomes, but current diagnostic methods remain largely invasive, highlighting the need for reliable non-invasive alternatives. Acoustic voice signals offer a non-invasive screening input, but their clinical value depends on whether signal-processing features and learned models can distinguish malignancy from acoustically similar benign lesions rather than merely separate pathological from healthy voices. Despite growing research on acoustic analysis combined with machine learning (ML) techniques, no systematic synthesis of this evidence currently exists. Therefore, the present review aimed to identify acoustic features that differentiate early glottic malignancies from both benign vocal fold lesions and normal vocal function using various ML techniques. **Method**: Following PRISMA 2020 guidelines, a comprehensive literature search was conducted across Scopus, EBSCO, Ovid (Embase), and PubMed databases through November 2025. Eighteen studies met inclusion criteria, and PROBAST was used for quality appraisal of included studies. Data extraction focused on acoustic feature categories, ML techniques, classification performance metrics, and validation strategies. **Results**: Nine acoustic feature categories emerged, with cepstral coefficients (particularly MFCCs) most prevalent across 55.6% of studies. Binary pathological-versus-normal classification achieved consistently high accuracy (96–98.3%) across both traditional machine learning and deep learning approaches. However, malignant-versus-benign discrimination demonstrated substantially lower performance (81–87.88% accuracy, AUC 0.631–0.91), with voice-only models proving insufficient. Multimodal models incorporating clinical variables or laryngoscopic images achieved higher performance, but their results cannot be attributed to voice features alone. All 17 prediction studies were judged at high overall risk of bias, predominantly because of small effective sample sizes, model-selection optimism, possible data leakage, and limited independent validation. **Conclusions**: ML can detect broad voice pathology, but current evidence is insufficient to support stand-alone acoustic screening for early glottic cancer. Future studies require clearly defined cancer-specific targets, patient-level analysis, standardized acquisition, nested model development, calibration, and independent multicentervalidation.

## 1. Introduction

Glottic carcinoma, arising from the vocal folds within the larynx, remains a pressing concern in global oncology, representing roughly two-thirds of laryngeal malignancies worldwide [1]. Epidemiological reports indicate that laryngeal cancer accounts for a substantial fraction of head and neck cancers worldwide, with over 180,000–200,000 new cases each year and marked regional disparities in incidence and survival [2,3,4]. Although early-stage tumors (T1–T2N0) generally have an excellent prognosis when diagnosed promptly, as patients often experience persistent hoarseness early in the disease course, even when tumors remain small [5]. This early warning signal creates a valuable opportunity for intervention, yet many individuals still receive their diagnoses at advanced stages when treatment becomes more complex and outcomes less favorable [6,7].

The challenge lies not in recognizing that voice changes matter, but in developing reliable, accessible methods to identify which voice alterations signal malignancy. Traditional diagnostic pathways depend heavily on laryngoscopy, imaging studies, and tissue sampling—all valuable tools, yet subjective and dependent on specialist availability [5]. For patients in underserved areas or regions with limited access to otolaryngologists, these barriers can mean delayed detection and missed opportunities for early treatment [8,9,10]. Given that voice-related symptoms often precede other clinical signs, strategies that harness voice as an accessible biological signal may be particularly valuable in low-resource or high-risk populations where specialist services are limited [11,12].

### 1.1. Significance of Voice-Based Screening

Voice production is remarkably sensitive to even subtle changes in vocal fold structure and function [13]. When early malignant transformation occurs, it disrupts normal vibration patterns and glottal closure mechanics in measurable ways [14]. These disruptions manifest as quantifiable acoustic changes that can be captured through relatively simple voice recordings. Quantitative voice assessment allows these deviations to be captured through perturbation metrics such as jitter and shimmer, noise-related measures including harmonics-to-noise ratio, fundamental frequency statistics, and formant-based indices [13,15,16,17].

Beyond traditional perturbation and spectral descriptors, feature sets derived from mel-frequency cepstral coefficients (MFCCs) and related cepstral representations have shown particular sensitivity to the subtle spectral–temporal changes characteristic of pathological voices [12,18,19]. These higher-level acoustic features have achieved robust performance in distinguishing laryngeal or glottic cancers from benign conditions and from other dysphonia, especially when combined with advanced classification algorithms [11,20,21,22]. Parallel work on vocal tract-related measures and nonlinear acoustic parameters has further expanded the repertoire of potential biomarkers, reinforcing the notion that carefully selected acoustic features can provide an informative window into early glottic pathology [23,24].

Over the past two decades, acoustic voice analysis has emerged as a promising adjunct to conventional laryngoscopic examination, offering advantages such as non-invasiveness, repeatability, and relatively low cost [17]. Itis objective, generating numerical measurements rather than subjective impressions. Due to its suitability for remote or community-based screening it can be performed in primary care settings, community health centers, or even through telemedicine platforms, bringing screening capabilities to populations who might never see a laryngologist [16]. Collectively, these findings support the concept of voice-based screening tools that could flag individuals who warrant more detailed otolaryngological evaluation.

### 1.2. The Role of Machine Learning

In tandem with these advances in acoustic analysis, artificial intelligence (AI) and machine learning (ML) have transformed approaches to pattern recognition in oncology and in voice-based diagnostics [25,26]. ML algorithms excel at finding patterns in complex, high-dimensional data, exactly the kind of data that acoustic voice analysis generates [25]. Traditional machine learning approaches have shown encouraging results in distinguishing healthy voices from those affected by laryngeal pathology [27]. Conventional supervised learning models, including classical classifiers and deep neural networks, have delivered encouraging results in distinguishing normal voices from pathological ones and in differentiating laryngeal cancer from other vocal disorders using audio recordings alone [11,19,28]. However, such approaches typically depend on expert-driven choices regarding feature extraction, model architecture and hyperparameter tuning, which can be time-consuming, require substantial technical expertise and may limit reproducibility across settings [24,28]. These constraints can pose barriers to widespread clinical adoption, particularly in environments where dedicated data science support is scarce [29,30].

In the present study, analytical approaches in this domain are classified into three distinct levels: (1) acoustic analysis, involving deterministic signal processing extraction (e.g., perturbation indices, MFCCs) without statistical classification; (2) conventional machine learning (ML), utilizing supervised algorithms (e.g., SVM, Random Forest) trained on handcrafted acoustic features; and (3) Deep Learning (DL), utilizing multi-layered neural networks (e.g., CNN, RNN) for joint representation learning and classification.

## 2. Need and Aim of the Review

Increased attention has been directed towards identifying sensitive acoustic biomarkers that are most informative for early-stage glottic cancer detection. Studies [27,31,32] have reported varying degrees of diagnostic accuracy using different acoustic feature sets and ML frameworks, highlighting the need for systematic synthesis of available evidence. Despite growing research in this domain, there is currently no comprehensive systematic review that critically evaluates the role of early detection of glottic cancers. So, the present study aimed to systematically characterize acoustic features that differentiate early glottic malignancies from both benign vocal fold lesions and normal vocal function using various ML techniques. The current review further assesses the performance of multiple ML frameworks in processing voice recordings for cancer classification, comparing their accuracy, reliability, and practical usability.

The work focuses specifically on identifying which acoustic features carry the strongest discriminative signals and evaluating how various ML platforms perform when applied to voice analysis tasks. By synthesizing current evidence, identifying methodological strengths and gaps, and highlighting clinically relevant acoustic biomarkers, this review seeks to inform future research and support the development of reliable, non-invasive screening tools for the early diagnosis of glottic malignancies.

## 3. Method

### 3.1. Study Design and Search Strategies

**Figure 1:**
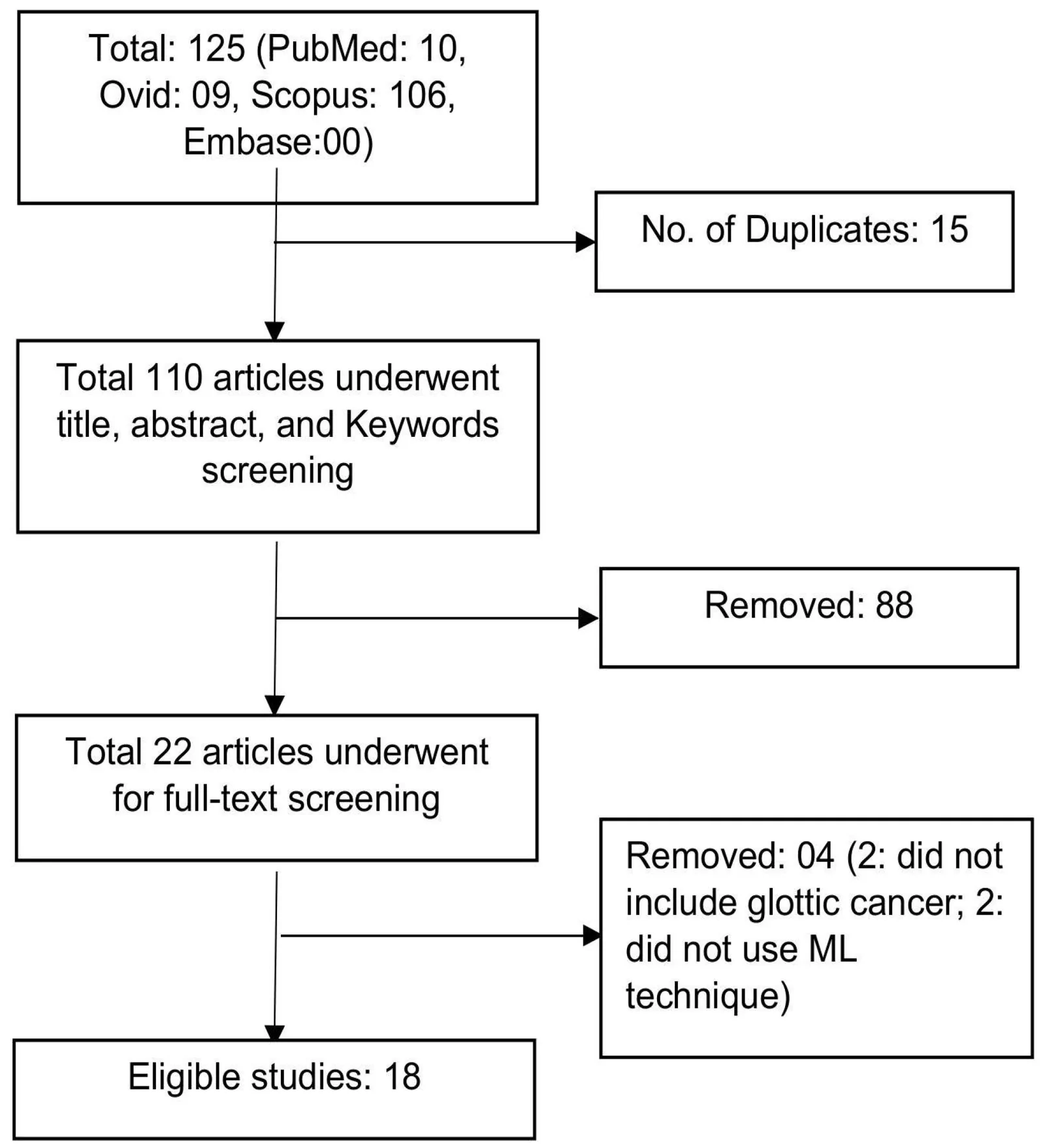
PRISMA Flowchart.

The current systematic review was registered with the Open Science Framework (OSF) with registration id osf.io/s4kep. The review was undertaken following the Preferred Reporting Items for Systematic Reviews and Meta-Analyses (PRISMA) guidelines outlined by Moher et al. [33]. The objective of this review was to critically examine and synthesize existing literature exploring the various acoustic parameters and ML techniques for the early detection of glottic cancer in ear. A comprehensive and systematic search of the literature was executed across the Scopus, EBSCO, Ovid (Embase), and PubMed databases, encompassing all relevant articles from their earliest entries until November 2025, with search verification updated prior to final synthesis. In the current review, database selection was restricted to these major structured bibliographic indices to ensure reproducible, Boolean-controlled search syntax and peer-reviewed coverage. Google Scholar was excluded due to lack of search string reproducibility and gray literature noise, while Web of Science was largely captured via Scopus and PubMed overlaps. Database hits in Figure 1 reflect deduplicated unique contributions.

The search strategy incorporated both Medical Subject Headings (MeSH) and relevant free-text terms, as detailed in Supplementary File S1. Additionally, the reference lists of the included studies were manually screened to identify any further eligible records.

### 3.2. Publication Selection

Following the initial database search, two reviewers (HV and RM) independently undertook the screening of all retrieved records. To ensure consistent application of the eligibility criteria, an a priori calibration exercise was conducted on a random 5% sample of the identified publications. Thereafter, both reviewers assessed records based on titles, keywords, and abstracts in line with the predefined inclusion and exclusion criteria, after which potentially eligible studies were subjected to full-text review.

Both the reviewers independently reviewed the records and marked as include, exclude, or uncertain decision against the prespecified criteria. Disagreements were resolved by discussion using the full-text eligibility rules over regular meetings. In cases where consensus could not be achieved, a third, senior reviewer (BN) adjudicated to provide the final decision on study inclusion. All citations and screening decisions were organized and managed using Microsoft Excel 360.

### 3.3. Eligibility Criteria

The Population, Concept, and Context (PCC) framework guided the formulation of inclusion criteria for this systematic review, as shown in Table 1. Eligible primary studies were published in English and evaluated acoustic voice data using a predictive ML or deep-learning model for one of four explicitly separated targets: general pathology versus normal voice, premalignant lesion detection, cancer versus healthy controls, or malignant versus benign lesion discrimination. In the current review, only manuscripts published in the English language were considered. To avoid duplication of data, inclusion was limited to original primary research studies. Review articles, case reports, editorials, and letters to the editor were excluded. Studies limited to the automated calculation of acoustic measures without a predictive model were excluded from model-performance synthesis; one PCA-based report was retained only as descriptive feature-level context and was not appraised with PROBAST. No limitations were imposed regarding the study design or participant characteristics. The present study included those studies which used the various ML techniques to analyze the various acoustic parameters for the early detection of the glottic cancer. Voice-only and multimodal studies were eligible only when a voice component was present. For multimodal studies using laryngoscopic images, electroglottography, demographics, questionnaires, or structured clinical records, modality-specific and fused-model results were extracted separately. Fused performance was not attributed to acoustic voice features alone. Confirmed cancer, carcinoma in situ, dysplasia or other premalignant lesions, unspecified tumors, and general voice pathology were coded as distinct target populations and were not treated as interchangeable evidence of early glottic-cancer detection.

### 3.4. Data Extraction

The data extraction instrument was designed in accordance with contemporary methodological guidelines. Calibration of the extraction template was performed using a 5% random subset of the included studies to verify its reliability and completeness. Key study details including title, authorship, participant numbers, type of ML technique, acoustic markers, gender distribution, primary outcomes, and other pertinent findings, were independently recorded by two reviewers (HV and RM) into Microsoft Excel (version 2010; Supplementary File S2) to mitigate extraction bias. Discrepancies arising during this process were reconciled via discussion during scheduled weekly team meetings. Persistent disagreements were adjudicated by a third, senior reviewer (BN). This iterative methodology facilitated continuous enhancement of the extraction framework across the review.

### 3.5. Quality and Risk of Bias Assessment

*Assessment framework:* The methodological robustness and risk of bias of all eligible studies were appraised using the Prediction model Risk Of Bias ASsessment Tool (PROBAST) [34], which is tailored for evaluations of multivariable prediction model studies. PROBAST was chosen because every included article focused on developing or validating machine learning–based prediction models for glottic cancer detection or classification. This instrument provides judgments on both risk of bias and concerns about applicability across four domains: Participants, Predictors, Outcome, and Analysis.

*Assessment procedure:* Two reviewers (HV and RM) independently applied the PROBAST to each study. Within every domain, they worked through the relevant questions to identify possible bias, and then assigned domain-level ratings of low, high, or unclear risk of bias.

An overall risk of bias classification (low, high, or unclear risk of bias) was subsequently derived for each study by integrating judgments across all four domains. Any study with at least one domain judged to be at high risk—especially the Analysis domain, where issues such as limited sample size, absence of external validation, or inappropriate handling of complex models could distort performance estimates—was categorized as having a high overall risk of bias. Discrepancies between the two reviewers were discussed in scheduled consensus meetings, and unresolved disagreements were adjudicated by a senior reviewer (BN). The results were summarized in tabular form to ensure transparent reporting of quality assessments.

*Key Assessment Criteria:* Within each PROBAST domain, the following key criteria were emphasized given their particular relevance to machine learning-based diagnostic prediction models:

Participants Domain: Sample size adequacy relative to the number of outcome events (malignancy cases), appropriateness of inclusion/exclusion criteria, potential for selection bias, matching of comparison groups, and representativeness of the study population for the intended clinical application.

Predictors Domain: Standardization of voice recording protocols including recording environment (soundproof booth versus clinical setting), equipment specifications, microphone-to-mouth distance, sampling rates, voice tasks performed, and consistency of acoustic feature extraction methods. Particular attention was paid to whether predictor assessments were made without knowledge of outcome data and whether all predictors would be available at the time the model is intended for clinical use.

Outcome Domain: Appropriateness of the reference standard (histopathological confirmation); use of pre-specified outcome definitions; clear differentiation between malignant, benign, and normal classifications; and independence of outcome determination from predictor measurements.

Analysis Domain: Assessment of sample size relative to model complexity (events-per-variable ratio), appropriateness of the validation strategy (internal cross-validation versus external validation on independent datasets), handling of missing data, prevention of data leakage between training and testing sets, completeness of performance metric reporting (including sensitivity, specificity, accuracy, AUC, confidence intervals, and calibration measures), and consideration of model overfitting risks. For deep learning architectures, the relationship between the number of trainable parameters and available training samples was specifically evaluated.

*Synthesis and reporting of quality assessments:* Risk-of-bias judgments were synthesized descriptively and visualized in summary tables showing domain-level and overall ratings for each study. The implications of observed biases for effect estimates and for the overall strength of the evidence were explicitly considered when interpreting results. Studies judged to have high risk of bias, especially those with very few malignancy cases (for example, fewer than 25) or without any form of external validation, were flagged, and their limitations were highlighted when considering reliability and generalizability of diagnostic performance metrics. These quality assessments informed confidence in the pooled findings and shaped recommendations for future research.

### 3.6. Synthesis of the Results

The findings were synthesized and reported in adherence to the updated PRISMA 2020 guidelines [35] (see Supplementary File S3). Studies were first classified as descriptive acoustic analysis, conventional ML, or deep learning according to the procedure reported. Results were then stratified by clinical target (general pathology versus normal, premalignant lesion detection, cancer versus normal, and malignant versus benign), input modality (voice only versus multimodal), unit of analysis, and validation design. Group-level acoustic differences were not combined with prediction-model performance, and internally validated estimates were distinguished from independent external validation.

## 4. Results

A comprehensive search across multiple electronic databases, a total of 125 records were retrieved. These records were processed according to the updated PRISMA 2020 guidelines, as detailed in Figure 1. Following the elimination of 15 duplicates, 110 records proceeded to title, abstract, and keyword screening, resulting in the exclusion of 88 records and advancement of 22 potentially eligible studies. Subsequent full-text evaluation of 22 articles led to the exclusion of four studies: two [36,37] did not include targeted population, i.e., glottic cancer, and two [19,38] did not use any ML technique to process the data.

### 4.1. Characteristics of Included Records

Most studies used cross-sectional (n = 10) study design, followed by retrospective (n = 5) designs, and three case–control studies. Sample sizes for malignancy groups demonstrated considerable heterogeneity, ranging from three cases [17] to 128 cases [39]. One computational study (Sachane and Patil [39]) analyzed 1320image patches extracted from 33 patients alongside an unlinked voice database, representing a patch-level rather than a patient-level cohort. Benign lesion groups were typically larger, ranging from 49 to 1940 participants, while control groups ranged from 25 to 350 healthy participants, as shown in Table 2. Publication years ranged from 2001 to 2025, with 61% (n = 11) published within the last five years, reflecting growing interest in conventional ML and deep learning applications for voice pathology detection. The Saarbrücken Voice Database (SVD) was the most used external dataset for validation or training.

Study populations included patients with histologically confirmed early glottic malignancies, predominantly squamous cell carcinoma (SCC), carcinoma in situ (CIS), dysplasia, and leukoplakia [18,40,41,42]. Benign vocal fold lesion groups comprised polyps, nodules, cysts, Reinke’s edema, vocal fold paralysis, and other phonotraumatic lesions [43,44,45]. Several studies exclusively included male participants due to higher laryngeal cancer prevalence in men [12,40], while others included mixed-gender populations [20,43,46]. Age distributions were generally matched between groups when reported, though Wang et al. [40] acknowledged their age- and sex-matched design may not be robust for younger and female patients.

**Table 2:** Characteristics of included studies.

Author and Year	Study Type	Study Population	Malignancy Sample	Benign/Other Sample	Control Sample
Gour et al., 2020 [12]	Case–control	Laryngeal cancer vs. healthy	55 (various laryngeal cancers)	None	55
Godino-Llorente et al., 2004 [17]	Cross-sectional	Pathological vs. normal voices	3 (carcinoma)	79 (polyps, nodules, cysts, others)	53
Song et al., 2024 [18]	Cross-sectional	Laryngeal cancer, benign, paralysis, healthy	30 (glottis/transglottic cancer)	81 (polyps, nodules, granulomas, Reinke’s edema)	155
Ben Aicha et al., 2024 [20]	Cross-sectional	Premalignant Lesions vs. normal	93 (leukoplakia, erythroplakia, keratosis)	None	54
Sachane et al., 2025 [39]	Computational	Cancerous vs. healthy	33	Not specified	259
Wang et al., 2022 [40]	Retrospectivecase–control	Glottic neoplasm vs. benign voice disorders	43 (dysplasia, CC, leukoplakia)	129 (vocal palsy, atrophy, phono traumatic lesions)	None
Kim et al., 2024 [42]	Retrospective	Laryngeal cancer, paralysis, benign, healthy	30 (SCC, early stage)	81 (reactive nodules, Reinke’ sedema)	155
Liu et al., 2025 [43]	Cross-sectional	Normal, benign, malignant	41 (glottic carcinoma)	64 (Reinke’s edema, polyps, cysts, leukoplakia)	52
Kim et al., 2025 [44]	Retrospective	UVFP, VF lesions, healthy	14 (SCC)	49 (polyps, cysts) + 105UVFP	41
Verikas et al., 2010 [45]	Cross-sectional	Cancerous, non-cancerous, healthy	22 (carcinoma)	193 (nodules, polyps, cysts, papillomata)	25
Mahmood et al., 2025 [46]	Cross-sectional	Pathological vs.healthy	128	445	350
Kwon et al., 2025 [47]	Cross-sectional	Cancer vs. non-cancer	62 (SCC)	Polyps (71), nodules (32), cysts	Included in non-cancer
Sahoo et al., 2025 [48]	Retrospective	Pathological vs. normal	Not specified separately	199 (polyps, cysts, paralysis)	177
Reid et al., 2022 [49]	Cross-sectional	Pathological vs. healthy	Not specified separately	Included in pathological	Included
Kanagachalam et al., 2025 [50]	Retrospective	Six-class pathology	46 (carcinoma, leukoplakia, tumors)	89 (laryngitis, vocal polyp)	90
Alonso et al., 2001 [51]	Case–control	Pathological vs. healthy	Carcinoma included	68 (nodules, polyps, Reinke’s edema)	100
You et al., 2025 [52]	Case–control	Tumor vs. non-tumor	71	Includes nodules, polyps, cysts	Included in non-tumor group

Note: VFL = vocal fold lesions; SCC = squamous cell carcinoma; CIS = carcinoma in situ; UVFP = unilateral vocal fold paralysis; VF = vocal fold.

### 4.2. Quality Assessment and Risk of Bias

#### 4.2.1. Overall Quality of Evidence

Risk of bias assessment using PROBAST revealed uniformly high risk of bias across the 17 assessable studies (Table 3). However, we were not able to score Liu et al., [43], as it used descriptive principalcomponent analysis of acoustic parameters rather than a prediction model. All 17 studies (100%) were rated overall High risk of bias, driven predominantly by the Analysis domain, in which every study (17/17, 100%) received a High rating owing to small malignancy sample sizes relative to model complexity, the absence of independent external validation, or optimism from feature-selection, dimensionality-reduction, or threshold-tuning performed on the same data used to report final performance. Wang et al. [41] was comparatively the lowest-concern study, combining pathologically confirmed outcomes with genuine independent external validation, yet it too received an Analysis-domain High rating because of a small malignant-case count relative to model complexity.

#### 4.2.2. Domain-Specific Risk of Bias Assessment

*Participants Domain*: Risk of bias was rated High for 15 of the 17 assessed studies, driven chiefly by small or narrowly defined malignant/cancer subgroups: Verikas et al. [45] included only 22 cancerous cases of 240 total, Kim et al. [44] pooled 14 internal and 5 external squamous cell carcinoma cases together with benign polyps and cysts under a single “VF lesion” label, and Godino-Llorente and Gómez-Vilda [17] reported carcinoma cases only briefly within a broader mixed-pathology cohort without stating their proportion—sample characteristics inadequate for reliable machine learning model development regardless of algorithmic sophistication. An additional concern involved participant representativeness, with several studies restricting enrollment to a single sex or a single center [12,42,47], and Sachane and Patil [39] combining two unlinked cohorts, a narrow-band laryngoscopy imaging arm and a general (non-cancer-specific) voice-pathology arm, with no patient-level linkage between modalities. Only Wang et al. [40] and Wang et al. [41] were rated Low risk in this domain, reflecting well-matched case–control or consecutively recruited, pathologically confirmed cohorts.

Multiple studies relied heavily on the Saarbrücken Voice Database (SVD), which consists predominantly of German speakers [46,49], while others drew on the MEEI database or pooled SVD and MEEI cohorts despite differing recording protocols [17,20,48]. Reid et al. [49] illustrated a related concern: their external clinical validation sample was very small (n = 30) and was recorded under materially different conditions (no soundproofing, a different microphone) from the SVD training data, introducing domain-shift risk between training and validation populations. The predominance of single-center studies further limited assessment of cross-population generalizability, with no multi-center studies identified in the review.

*Predictors Domain:* The Predictors domain was rated a Low risk of bias for 16 of the 17 assessed studies, reflecting largely standardized voice-recording protocols; Kim et al. [44] was the exception, rated Unclear because its two participating institutions used different microphones and hardware without controlling mouth-to-microphone distance. Recording environments nonetheless varied across the wider literature, ranging from professionally soundproofed acoustic booths to standard clinic rooms without formal noise control, which introduces potential measurement error in acoustic features, particularly for noise-related parameters such as harmonics-to-noise ratio (HNR) and normalized noise energy (NNE) that are inherently sensitive to background acoustic conditions.

Sampling rates varied considerably across studies (22,050 Hz to 50,000 Hz), potentially affecting the capture of high-frequency acoustic information relevant to glottic pathology. While most studies employed the sustained vowel /a/ as the primary voice task, offering good reproducibility, several studies provided insufficient detail regarding microphone specifications, microphone-to-mouth distance standardization, or whether recording personnel were blinded to participant diagnostic status. These methodological gaps introduce uncertainty about predictor measurement quality and potential information bias.

Studies utilizing standardized databases such as SVD demonstrated lower risk of bias in this domain due to controlled recording conditions and systematic acoustic feature extraction. However, the clinical applicability of such highly controlled recordings to real-world implementation scenarios, where recordings may occur in varied clinical environments with heterogeneous equipment, remains a moderate concern.

*Outcome Domain:* The Outcome domain showed the widest spread of ratings of the four PROBAST domains: 6 of 17 studies were rated Low risk, 9 were rated Unclear, and 2 were rated High risk. Where histopathological examination was clearly used as the reference standard for malignancy and outcome categories were unambiguously specified, risk of bias was Low; incorporation bias was minimized where diagnoses were established independently of acoustic voice analysis.

The two High ratings reflect the most serious outcome-definition concerns in the set. Sahoo et al. [48] framed its pipeline as “laryngeal cancer” detection, yet the outcome labels used were, on inspection, general voice-disorder diagnoses (dysphonia subtypes, laryngitis, vocal-fold paralysis) without histological or clinical confirmation of malignancy anywhere in the pipeline. Kanagachalam and Kim [50] pooled vocal-cord carcinoma with leukoplakia, a premalignant, non-malignant lesion, and unspecified “tumors” under a single “organic-structural (malignant)” class without histological differentiation, and this class was never independently evaluated in held-out testing. The nine Unclear ratings generally reflected incomplete specification of outcome determination procedures or unclear composition of pathological versus normal groups [12,17,18,20,39,44,46,51,52]. For studies examining premalignant lesions such as leukoplakia or dysplasia, the potential for diagnostic uncertainty in distinguishing early malignant transformation from severe dysplasia introduces some degree of outcome misclassification risk, though this likely reflects genuine clinical diagnostic challenges rather than methodological flaws.

*Analysis Domain*: The Analysis domain was the single most consistent source of bias in the review. Every one of the 17 assessed studies was rated High risk. Three critical issues were identified. Sample Size Inadequacy: A fundamental concern involved the substantial mismatch between sample sizes and model complexity across nearly all studies. Malignant/cancer sample sizes were as small as 14–22 cases in some studies [44,45]. Wang et al. [41], supported a comparatively complex CNN-BiGRU-attention architecture with only 60 primary and 23 external malignant cases. Traditional guidance suggests a minimum of 10–20 events (outcome cases) per predictor variable for conventional regression models, while deep learning architectures with thousands or millions of trainable parameters theoretically require hundreds to thousands of outcome events to prevent overfitting.

Studies employing deep neural networks, convolutional neural networks, or recurrent architectures with small malignancy samples (n < 50) faced substantial overfitting risk. For instance, Song et al. [18] reported 100% classification accuracy for cancer detection using a CNN architecture with only 30 malignancy cases, a result that strongly suggests model memorization rather than genuine pattern learning. Similarly, Kim et al. [44] applied deep learning methods to only 14 squamous cell carcinoma cases, yielding model performance metrics of questionable reliability despite sophisticated analytical approaches.

Validation Strategy Limitations: A critical weakness involved the predominance of internal validation strategies without external testing. Only Wang et al. [41] performed formal external validation on independent datasets, population, and language, reproducing a comparable AUC to its primary cohort (0.785 vs. 0.807–0.878). Kim et al. [44] also tested on an external cohort but saw performance collapse there. The remaining studies relied exclusively on internal cross-validation or hold-out validation using data splits from the same source population or reused the same small sample to both tune and evaluate the model [47]. Internal validation approaches, particularly when applied to small datasets, are known to overestimate model generalization performance. Where external validation was attempted, it often revealed concerning performance degradation. Kim et al. [44], despite genuine external testing, saw multiclass accuracy fall to 35–45% on the external cohort versus much higher internal figures, confirming substantial overfitting in the internally validated model. Even Wang et al. [41], comparatively the strongest-designed study, reported that voice signal alone was insufficient for discrimination (AUC 0.631 primary/0.470 external), with acceptable being performance reached only after demographic and structured clinical data were added. These findings suggest that many reported performance metrics in this literature likely represent optimistic estimates that would not be replicated in real-world clinical implementation.

Incomplete Performance Reporting and Same Data Tuning: Across most studies, feature selection, dimensionality reduction, or classification-threshold tuning were performed on the same data later used to report final performance, without an independent held-out test set—for example, genetic-search feature selection and SVM tuning in Verikas et al. [45], forward feature selection and grid search in Gour et al. [12], and a posthoc classification threshold in Godino-Llorente and Gómez-Vilda [17]. Reporting completeness also varied substantially: while accuracy was almost universally reported, critical metrics for clinical decision-making were frequently absent. Sensitivity and specificity, essential for understanding the balance between cancer detection and false positive rates, were marked as “not reported” (NR) for multiple studies in the systematic review’s performance tables. Area under the receiver operating characteristic curve (AUC), which provides a threshold-independent assessment of discriminative ability, was missing for a substantial proportion of studies. Confidence intervals or standard errors for performance estimates were rarely reported, preventing assessment of statistical precision and making it impossible to determine whether performance differences between methods were meaningful or simply reflected sampling variation. No studies reported calibration metrics, which assess the agreement between predicted probabilities and observed outcome frequencies—a critical consideration for clinical risk-prediction tools. The absence of calibration assessment means that even models with high discrimination (AUC) may produce poorly calibrated probability estimates unsuitable for individual patient counseling.

#### 4.2.3. Applicability Concerns

Beyond risk of bias, PROBAST assessment identified several applicability concerns that may limit the translation of study findings to routine clinical practice:

*Population Applicability*: Studies with male-only recruitment [12,42,47] demonstrated high concern for applicability to general clinical populations where both sexes require screening. The predominance of single-center studies and heavy reliance on the Saarbrücken Voice Database raised concerns about applicability across diverse healthcare settings, linguistic groups, and populations with varying baseline laryngeal cancer risk profiles. Sachane and Patil [39] raised a distinct applicability concern: their federated pipeline combined an imaging cohort of laryngoscopy patients with a voice cohort of general, non-cancer-specific pathological voices, so the reported “voice” performance does not represent a confirmed cancer population.

*Predictor Applicability*: Studies employing highly controlled recording environments with professional-grade equipment raised high concerns regarding practical implementation feasibility. The requirement for soundproof acoustic booths, specialized microphones, and technically trained recording personnel may not be achievable in primary care settings, community health centers, or telemedicine platforms, precisely the contexts where accessible voice-based screening would provide greatest value for early detection. Reid et al. [49] underscored this concern directly: their model was trained on soundproofed, headset-microphone recordings but evaluated on non-soundproofed clinic recordings from a different microphone, a domain shift that likely contributed to wide, unstable confidence intervals on external performance. The heterogeneity in recording protocols across studies also suggests that standardized acquisition procedures would need to be established before clinical deployment.

Model Complexity Applicability: Studies implementing sophisticated deep learning architectures raised concerns regarding computational resource requirements and technical expertise needed for deployment. Convolutional neural networks, recurrent architectures with attention mechanisms, and transfer learning approaches require substantial computational infrastructure and specialized programming knowledge that may not be available in resource-limited clinical settings. Sachane and Patil [39] illustrate the extreme end of this concern, pairing a hybrid quantum-dilated-CNN/deepneuro-fuzzy architecture with a very small patient-level imaging sample (33 patients).

### 4.3. Acoustic Feature Categories

Nine distinct categories of acoustic features were identified across the included studies. The most prevalent feature category was cepstral coefficients, utilized in 10 of 18 studies (55.6%), including mel-frequency cepstral coefficients (MFCCs), delta MFCCs, linear predictive cepstral coefficients (LPCCs), and bark-frequency cepstral coefficients (BFCCs) [12,17,18,20,39,40,42,44,48,52], as shown in Table 4.

Frequency measures constituted the second most common category, including fundamental frequency (F0), formant frequencies (F1–F4), and formant dispersion, examined in four studies [20,43,44]. Perturbation measures (jitter, shimmer, amplitude perturbation quotient, pitch perturbation quotient) were analyzed in four studies [17,20,44,45], while noise measures (harmonics-to-noise ratio, normalized noise energy, cepstral peak prominence) appeared in four studies [17,20,43,45].

Advanced feature extraction approaches included spectral features (spectral centroid, spectral spread, spectral skewness, root mean square error) in two studies [39,43], time–frequency representations (mel-spectrograms, Gammatonegram, TKEO-Scalogram, CGT-Scalogram) in three studies [46,49,50], nonlinear features (mutual information, correlation dimension, Renyi entropy) in one study [12], higher-order statistics (bicoherence indices, kurtosis interference) in one study [51], and novel filter approaches (octave frequency spectrum energy) in one study [18].

### 4.4. Voice Recording Parameters and Protocols

The sustained vowel/a/was the most common voice task across studies, typically recorded for 2–5 s [18,20,40,42,43]. Sampling rates varied considerably, ranging from 22,050 Hz to 50,000 Hz [40,42,43]. Recording environments included sound-proof booths, clinical settings with controlled acoustics, and clinic rooms without formal soundproofing, contributing to variability in acoustic quality [44,52].

Several studies utilized the Saarbrücken Voice Database (SVD), a publicly available dataset consisting predominantly of German speakers, for external validation or training purposes [17,46,49]. Recording equipment ranged from professional-grade microphones in controlled environments to standard clinical recording devices, with microphone-to-mouth distances typically maintained at 10–30 cm when specified.

### 4.5. Specific Acoustic Parameters Discriminating Pathology Types

Liu and colleagues’ [43] study employed principal component analysis to reveal unique voice patterns across different laryngeal conditions. Their findings showed that variability in root mean square error measurements was distinctive for healthy vocal production. For non-cancerous growths, they observed that fluctuations in the voice’s baseline pitch served as a key differentiator. Cancer-affected voices demonstrated elevated values in the third formant alongside greater variability in the fourth formant. The research also documented dispersed formant patterns in cases of Reinke’s edema, whereas polyp presentations were marked by notable measurements in the relationship between subharmonic and harmonic amplitudes.

Ben Aicha et al. [20] demonstrated that pitch (F0) exhibited the highest mutual information value (0.509) for detecting precancerous lesions, followed by spectral sub-band centroids (SSCs). Their system achieved 94% accuracy, 95% recall, and 89% precision for premalignant lesion detection using these features [20]. The importance of formant parameters was independently confirmed by Kim et al. [44], who identified Formant 1–3 parameters and MFCCs as top differentiating features through Kernel SHAP analysis.

Song et al. [18] demonstrated that octave frequency spectrum energy (OFSE) outperformed traditional MFCCs in all classification tasks. The over-formant frequency area (above 4 kHz) was particularly discriminative for laryngeal cancer versus healthy voices, utilized in 48% of classification decisions. In contrast, formant and fundamental frequency areas were more critical for differentiating benign mucosal disease and vocal fold paralysis.

### 4.6. Classification Performance: Binary Pathological vs. Normal

Five studies examined binary classification between pathological and normal voices, achieving consistently highperformance metrics. Table 5 presents detailed performance metrics for binary pathological versus normal classification. Godino-Llorente and Gómez-Vilda [17] employed learning vector quantization (LVQ) neural networks to achieve 96% frame accuracy with no misclassified records. Reid et al. [49] utilized visual geometry group (VGG19) convolutional neural network combined with support vector machine, achieving 97.8% accuracy, 98.5% sensitivity, and 97.1% specificity, with clinical validation maintaining 96.7% accuracy.

Sahoo et al. [50] implemented an iterative multi-level convolutional neural network (IML-CNN) achieving 97.36% accuracy with 95.36% recall and 96.25% F1-score. Mahmood et al. [46] employed ResNet architecture with combined Gammatonegram and TKEO-Scalogram features (CGT-Scalogram), achieving 96% accuracy, 96.1% recall, and 96.3% precision. Alonso et al. [51] combined classic acoustic parameters with higher-order statistics (HOS) parameters using neural networks, achieving 98.3% accuracy, representing the highest performance among binary pathological-normal classifiers.

### 4.7. Classification Performance: Binary Malignant vs. Benign

Performance depended strongly on the comparison group and input modality. For the clinically relevant malignant-versus-benign task, voice-based models reported accuracies from 81.5% to 87.88% and AUCs from 0.631 to 0.91. Kwon et al. [47] reported 87.88% for voice alone, 89.06% for laryngoscopic images alone, and 95.31% for image-plus-voice fusion. The fused estimate is presented as multimodal performance and is not evidence of acoustic-feature performance alone. Song et al. [18] compared cancer with healthy voices and is therefore summarized separately from malignant-versus-benign discrimination, despite its higher reported accuracy.

Subsequently, Wang et al. [44] revealed the fundamental significance of multimodal data fusion in diagnostic classification. Acoustic signal analysis as an isolated modality yielded an AUC of merely 0.631 for differentiating neoplastic from non-neoplastic glottic disorders, indicating inadequate discriminatory power. The incorporation of demographic variables enhanced model performance to an AUC of 0.807, whereas the integration of comprehensive structured clinical documentation—encompassing symptomatic presentations, comorbid conditions, and tobacco exposure history—through a hybrid CNN-GRU-Attention architectural framework achieved 81.5% accuracy, 78.3% sensitivity, 81.6% specificity, and 0.878 AUC, representing a 39% augmentation in discriminative capacity relative to unimodal acoustic analysis.

Song et al. [18] reported optimal performance metrics for malignancy detection through the implementation of convolutional neural network architectures utilizing octave frequency spectrum energy feature extraction, achieving 100% classification accuracy and 0.99 AUC when distinguishing normophonic voices from laryngeal carcinoma cases. Nevertheless, these exceptional performance outcomes were constrained to binary classification paradigms comparing healthy and malignant states, precluding the differentiation of intermediate benign lesion categories, as shown in Table 6.

### 4.8. Classification Performance: Multi-Class Pathology Differentiation

Multi-class classification consistently underperformed binary approaches across all studies, as shown in Table 7. Kim et al. [42] attempted four-class classification (healthy, laryngeal cancer, vocal fold paralysis, benign mucosal disease), achieving 75–83% accuracy depending on the specific algorithm implementation. Binary classification variants of the same study achieved substantially higher performance, ranging from 85 to 97%, highlighting the challenge of simultaneously differentiating multiple pathology type. You et al. [52] reported 94.37% accuracy for binary tumor versus non-tumor classification with 78.57% sensitivity and 98.25% specificity. However, when expanding to three-class classification (normal, benign, tumor), accuracy dropped to 75%, demonstrating the substantial performance degradation associated with increased classification complexity.

Kim et al. [44] examined the three-way classification of healthy vocal folds (HVFs), unilateral vocal fold paralysis (UVFP), and vocal fold lesions, achieving only 40% accuracy. Binary classifier variants in the same study reached 83% accuracy, representing a 107.5% relative improvement over the multi-class approach. Mahmood et al. [46] achieved 94.4% accuracy for multi-class pathology classification with 94.5% precision and 94% recall, though specific pathology categories were not clearly delineated. Verikas et al. [45] examined three-class classification (healthy, cancerous, noncancerous) achieving greater than 92% accuracy. Notably, they found that questionnaire data regarding patient symptoms and medical history were superior to voice features alone for classification purposes, with the cancerous class of 22 subjects limiting classification reliability. Gour et al. [12] achieved 80% accuracy for binary laryngeal cancer versus healthy classification using random forest classifier with nonlinear features including mutual information, correlation dimension, and Renyi entropy.

### 4.9. Database Dependencies and Cross-Linguistic Considerations

Multiple studies relied on the Saarbrücken Voice Database (SVD), which consists predominantly of German speakers, for algorithm training or validation [46,49], whereas some used the Mssachusetts Eye and Ear Infirmary (MEEI) database [17].Reid et al. [49] explicitly acknowledged uncertainty about whether acoustic properties remain consistent across languages, noting this as a limitation for generalizability to non-German-speaking populations.

The predominant use of single databases or institution-specific datasets limited assessment of cross-population generalizability. Only four studies (22.2%) conducted formal external validation on independent datasets [41,44,47,49], while the remaining 14 studies (77.8%) relied exclusively on internal cross-validation approaches that may overestimate true generalization capabilities.

### 4.10. Overview of Computational Approaches

Seventeen reports developed or evaluated predictive classifiers using conventional ML or deep learning. The PCA-based descriptive acoustic study was summarized separately and is not counted as a prediction model (Table 8).

Traditional machine learning approaches included learning vector quantization (LVQ) neural networks [17], support vector machines (SVMs) [49], ensemble decision trees [49], random forest classifiers [12], and conventional neural networks with higher-order statistics [51].

Deep learning implementations encompassed various architectures: convolutional neural networks (CNNs) alone [18,48], recurrent architectures with attention mechanisms [41], hybrid models combining CNN-GRU-Attention [41], transfer learning with VGG19 [49], ResNet variants [46], and iterative multi-level CNN architectures [48]. Deep neural networks (DNNs) with various configurations were employed by Wang et al. [40] and other investigators.

### 4.11. Traditional Machine Learning vs. Deep Learning for Binary Pathological-Normal Classification

Traditional machine learning approaches achieved comparable performance to deep learning methods for binary pathological-normal classification. Learning vector quantization achieved 96% accuracy [17], while neural networks with higher-order statistics achieved 98.3% accuracy [51]. These results were statistically indistinguishable from deep learning approaches: VGG19-SVM achieved 97.8% [49], iterative multi-level CNN achieved 97.36% [48], and ResNet achieved 96% [46], as shown in Table 9.

### 4.12. Performance by Specific Algorithm and Architecture

Detailed performance metrics organized by specific algorithm architectures is presented in Table 10.

### 4.13. Deep Neural Network Variants

Wang et al. [40] employed deep neural networks with MFCC features achieving 86.11% accuracy for malignant–benign differentiation. The same research group later implemented CNN-GRU-Attention architecture incorporating multimodal data (voice, demographics, medical records), achieving 81.5% accuracy with 0.878 AUC. The recurrent architecture with attention mechanisms enabled processing of sequential acoustic patterns and integration of heterogeneous data types.

### 4.14. Convolutional Neural Network Architectures

Transfer learning with VGG19 pretrained on ImageNet, fine-tuned for voice spectrograms, achieved 97.8% accuracy with 98.5% sensitivity and 97.1% specificity [49]. Iterative multi-level CNN incorporating progressive feature refinement achieved 97.36% accuracy with 96.25% F1-score [48]. ResNet architecture processing combined Gammatonegram and TKEO-Scalogram time–frequency representations achieved 96% accuracy with 96.3% precision [46].

Song et al. [18] implemented CNN specifically optimized for octave frequency spectrum energy features, achieving 100% accuracy and 0.99 AUC for healthy-cancer classification. The architecture incorporated frequency-band-specific processing aligned with OFSE feature extraction methodology.

### 4.15. Ensemble and Multimodal Approaches

Kwon et al. [47] implemented ensemble decision trees combining predictions from laryngeal image classifiers and voice acoustic classifiers. The ensemble approach achieved 95.31% accuracy with 97.06% sensitivity and 93.33% specificity, substantially outperforming individual modalities: image-only (89.06%) and voice-only (87.88%) classifiers. The ensemble method employed weighted voting based on individual classifier confidence scores. Verikas et al. [45] compared multiple classifier types (neural networks, support vector machines, decision trees) and found that the integration of questionnaire data with acoustic features consistently outperformed acoustic features alone, achieving greater than 92% accuracy across three pathology classes.

## 5. Discussion

The evidence shows that acoustic voice data combined with ML can identify broad voice pathology, but it does not establish consistently accurate early glottic-cancer detection. Reported performance was highest for pathological-versus-normal and cancer-versus-healthy comparisons and lower for malignant-versus-benign or multiclass tasks. Conventional ML and deep learning were the dominant analytical approaches. Small, heterogeneous datasets, target-label inconsistency, and limited independent validation constrain generalizability. The review demonstrates that both traditional machine learning and deep learning achieve very high accuracy (often ≥96%) for the binary classification of pathological versus normal voices, indicating that acoustic alterations associated with laryngeal pathology are robust and machine-detectable. For example, LVQ and higher-order-statistics-based neural networks reached 96–98.3% accuracy, comparable to VGG19-SVM, iterative multi-level CNN, and ResNet architectures, underscoring that model family is less critical than careful feature engineering and preprocessing in this comparatively simple task.

In contrast, voice-based discrimination between glottic malignancies and benign vocal fold lesions was more challenging, with reported accuracies around 81.5–87.88% and clear reductions in sensitivity. Wang and colleagues [41] show that voice-only models achieve an AUC of 0.631, whereas adding demographics, clinical variables, and medical records increases AUC to 0.878, highlighting the limited standalone discriminative power of acoustic features for malignancy vs. benign separation and the value of multimodal fusion. Kwon et al. [47] reported 87.88% accuracy for voice alone and 95.31% after combining voice with laryngoscopic images. These gains support multimodal prediction but cannot be attributed solely to acoustic characteristics.

Multi-class classification (e.g., healthy vs. benign vs. cancer; or several laryngeal pathologies) consistently underperforms binary paradigms, with accuracies often falling to 40–83%. These findings emphasize that while voice carries strong signals of “any pathology,” fine distinctions remain difficult, particularly when benign and malignant lesions share overlapping acoustic manifestations or when class imbalance and small malignant cohorts constrain model learning.

### 5.1. Methodological Quality and Evidence Strength

Quality assessment using the PROBAST framework reveals that while acoustic voice analysis shows technical promise for laryngeal cancer detection, the current evidence base contains substantial methodological weaknesses that limit confidence in reported outcomes and prevent clinical adoption. All included studies were rated overall High risk of bias, with the Analysis domain, chiefly sample-size/model-complexity mismatch, absent or collapsing external validation, and same-data feature/threshold tuning.

### 5.2. Critical Sample Size-Complexity Mismatch

The most pervasive weakness involves applying sophisticated algorithms to datasets with insufficient malignancy examples. Typical studies include approximately 40 cancer patients yet employ neural network architectures containing millions of adjustable parameters, creating conditions where systems memorize training examples rather than learn authentic diagnostic patterns. Several publications report perfect classification despite analyzing fewer than 30 malignancy cases, results suggesting the capture of dataset idiosyncrasies rather than disease signatures. Traditional statistical guidance recommends at least 10–20 outcome examples per predictor variable, yet numerous deep learning implementations dramatically reverse this fundamental relationship.

### 5.3. External Validation Gap and Performance Optimism

Most reports relied on internal cross-validation or hold-out splits from the same source population, which do not test robustness to changes in patients, languages, equipment, or recording protocols. A small number evaluated an independent cohort or database, but the scope of validation differed. In Wang et al. [41], independent external validation was demonstrated for the acoustic and demographic sub-models (with voice-only AUC declining to 0.470 on the external cohort), whereas the full model incorporating structured medical records was evaluated only on internal hospital data due to a lack of standardized EHR structures at the external site.

External results should therefore be interpreted at the level of the specific model and modality that was tested rather than generalized to the complete pipeline. Reliance on predominantly German-language public voice databases further limits conclusions about cross-linguistic transportability.

### 5.4. Predictor Measurement Heterogeneity

Recording protocols vary dramatically across studies, from acoustically controlled laboratories to standard clinic rooms without noise management, introducing systematic measurement variability that confounds performance comparisons. Sampling rates range from 22,050 to 50,000 samples per second, affecting capture of high-frequency vocal information potentially relevant to early pathology detection. These inconsistencies raise questions about whether performance differences reflect genuine algorithmic advantages versus recording methodology artifacts, complicating reproducibility and data pooling efforts.

### 5.5. Incomplete Performance Reporting and Clinical Utility Uncertainty

Substantial gaps exist in outcome measurement transparency. While accuracy appears universally, sensitivity and specificity are frequently absent, preventing assessment of whether systems achieve the high sensitivity needed for screening or adequate specificity to avoid overwhelming referral pathways. Most critically, no studies assess calibration, the agreement between predicted probabilities and observed outcomes, which is essential for patient counseling and shared decision-making. Even high-discriminating systems may generate poorly calibrated risk estimates unsuitable for individual communication.

### 5.6. Acoustic Biomarkers and Pathophysiological Relevance

Across the included studies, cepstral coefficients, particularly MFCCs and related variants, had emerged as the most frequently used and most informative features, supporting their status as core biomarkers for early glottic disease. This aligns with prior work showing that MFCCs and other cepstral measures capture subtle spectral–temporal perturbations arising from altered vibratory patterns and glottal closure mechanics in early malignancy.

The review also highlights the discriminative value of frequency-based measures (F0, formants, formant dispersion), perturbation indices (jitter, shimmer, APQ, PPQ), and noise measures (HNR, NNE, CPP), which collectively reflect irregularity, breathiness, and turbulence linked to structural vocal fold changes. Liu et al. [45] and Ben Aicha et al. [20] provide converging evidence that formant-related parameters and F0 carry high mutual information for differentiating precancerous or malignant states from benign and normal voices, while Song et al. show that OFSE, particularly over-formant frequency bands above 4 kHz, can outperform traditional MFCCs for cancer–healthy discrimination.

Nonlinear and higher-order statistical features (e.g., mutual information, correlation dimension, Renyi entropy, bicoherence indices) appear in only a minority of studies but show potential as complementary markers that capture complex dynamical behaviors not reflected in conventional linear metrics. However, the current evidence base is too limited to determine whether these features generalize across languages, recording setups, and pathology spectra, highlighting an important area for future methodological work.

### 5.7. Methodological Strengths and Limitations of the Evidence

A major strength of the included literature is the diversity of computational approaches, spanning traditional ML (SVM, LVQ, random forest, ensemble decision trees) and a range of deep architectures (standard CNNs, transfer learning, ResNet variants, CNN–GRU–Attention, Siamese networks, few-shot learning). This breadth allows robust cross-comparison and reveals that, for simpler binary tasks, sophisticated deep models do not necessarily yield large performance gains over well-tuned traditional methods, which may be easier to implement in resource-limited clinical environments.

Nonetheless, the evidence is limited by small and heterogeneous malignancy cohorts; wide variation in benign and control sample sizes; and inconsistent reporting of key methodological details such as recording environments, microphone specifications, and exact task protocols. Voice tasks were predominantly sustained /a/ produced for 2–5 s at varied sampling rates (22,050–50,000 Hz), with recordings acquired in settings ranging from sound-proof booths to ordinary clinic rooms, likely introducing uncontrolled acoustic variability that could bias feature distributions and model performance.

Database dependency and restricted external validation substantially limit generalizability. Many studies relied on the Saarbrücken Voice Database or single-institution datasets, and only about one-fifth performed formal external validation on independent cohorts. Reid et al. [49] explicitly noted uncertainty about cross-linguistic stability of acoustic markers, raising important questions about how language, phonetic structure, and cultural voice use patterns may affect both feature behavior and model calibration.

### 5.8. Clinical and Translational Implications

From a clinical standpoint, the high accuracy of pathological-versus-normal classification suggests that voice-based pre-screening tools could act as low-cost front-end triage systems to flag individuals who warrant laryngoscopic evaluation, especially in underserved or telehealth contexts. Such tools could be deployed in primary care, community screening programs, or smartphone-based applications, helping to address diagnostic delays that contribute to late-stage presentation and poorer survival in glottic cancer. However, the relatively modest performance of malignancy–benign and multi-class models indicates that acoustic analysis alone should not be used to rule out cancer or to guide definitive management decisions. The multimodal results from Wang et al. [41] and Kwon et al., [47] underscore that integration of voice with structured clinical data, demographics, and laryngoscopic imaging is likely necessary to achieve the discrimination required for safe, clinically actionable decision support in early glottic cancer.

The heterogeneity of feature sets and architectures across studies also has implementation consequences. While sophisticated models like CNN–GRU–Attention, OFSE-based CNNs, and Siamese networks may provide performance or sample-efficiency advantages, they demand greater computational resources and expertise, which may not be available in low-resource settings. Regardless of model family, clinical translation requires transparent preprocessing, leakage-resistant feature selection and tuning, calibration, and external validation. AutoML could eventually assist model development, but the included studies should not be labeled AutoML unless they automate substantial parts of preprocessing, feature or algorithm selection, hyperparameter optimization, and final model selection within an appropriate validation design.

### 5.9. Analytical Terminology and Future Model Development

The review’s focus on ML also resonates with broader trends in oncology and digital health. The reviewed evidence primarily concerns automated classification using conventional ML and deep learning. ML models learn a prediction function from labeled data; and deep-learning models may jointly learn representations and predictions. Maintaining these distinctions prevents group-level acoustic differences, internally validated classifiers, and externally validated prediction models from being interpreted as equivalent evidence. Translating these successes to voice-based glottic cancer screening will require careful curation of training data; explicit handling of class imbalance; and embedded mechanisms for monitoring model drift as recording hardware, languages, and clinical populations evolve.

Future work should prioritize the following:

- Larger, multicentric, and linguistically diverse datasets with standardized recording protocols and detailed metadata, enabling robust external validation and subgroup analyses.
- Prospective studies that integrate rigorously validated ML-based voice analysis into real-world clinical pathways, measuring not only diagnostic performance but also impacts on time-to-diagnosis, referral patterns, and patient outcomes.
- Transparent reporting and interpretability tools (e.g., SHAP, feature importance) to help clinicians understand which acoustic and non-acoustic factors drive predictions, thereby fostering trust and facilitating responsible adoption.

Collectively, the reviewed evidence supports the feasibility of using acoustic voice analysis with conventional ML and deep learning as a non-invasive adjunct for early glottic cancer detection. It does not yet support an autonomous screening system. Future implementation studies should preserve the distinction between voice-only and multimodal evidence, use cancer-specific clinical targets, and validate the complete locked pipeline before deployment.

## 6. Conclusions

Acoustic voice analysis combined with conventional ML or deep learning is promising for detecting broad voice pathology, but current evidence does not support stand-alone diagnosis of early glottic cancer. Malignant and benign lesions have overlapping acoustic signatures, and apparent performance depends strongly on the comparison group, unit of analysis, validation design, and use of non-voice inputs. Multimodal models may improve discrimination, but their performance cannot be attributed to voice alone. Progress requires cancer-specific target definitions, patient-level reporting, standardized and linguistically diverse datasets, leakage-resistant model development, calibration, and independent multi-center validation. Acoustic ML should therefore be positioned as decision support within a comprehensive diagnostic pathway rather than as a replacement for laryngoscopic and histopathological assessment.

## Figures and Tables

**Table 1:** Demonstrating the PCC framework.

**Population**	Individuals diagnosed with glottic (laryngeal) cancer
**Concept**	ML algorithms applied to acoustic voice analysis features
**Context**	Clinical or research settings

**Table 3:** PROBAST risk of bias assessment summary.

Study	Participants	Predictors	Outcome	Analysis	Overall ROB	Key Concerns
Gour et al., 2020 [12]	High	Low	Unclear	High	High	Small sample with laryngeal-cancer sites broader than glottic cancer alone; forward feature selection and SVM/RF hyperparameter grid search performed with very low events-per-variable, single train/test split, no independent external validation; histological vs. clinical confirmation of cancer diagnosis not explicitly stated.
Godino-Llorente & Gómez-Vilda, 2004 [17]	High	Low	Unclear	High	High	MEEI mixed-pathology cohort, limiting applicability to glottic cancer specifically; frame-level split raises data-leakage risk, since frames from the same recording/patient could appear in both training and test sets.
Song, Kim & Lee, 2024 [18]	High	Low	Unclear	High	High	Single-center, retrospective, male-only cohort; laryngeal cancer group small, no independent external validation cohort; histological confirmation of cancer cases implied but not explicitly stated.
Ben Aicha & Kacem, 2024 [20]	High	Low	Unclear	High	High	Two databases fused despite differing recording protocols; frame-level (not patient-level) analysis on 12,816 overlapping frames with 1354 duplicate lines noted raises data-leakage risk between train/validation/test, KPCA dimensionality reduction, and SVM hyperparameter grid search appear to be performed on the whole dataset before/alongside the final 70/30 split; very small precancerous subgroups.
Sachane & Patil, 2025 [39]	High	Low	Unclear	High	High	Federated “image + voice” pipeline combines two unrelated cohorts, 33 narrow-band laryngoscopy patients and the Saarbrücken Voice Database, with no patient-level linkage between modalities, so the “voice” case definition does not represent confirmed cancer. No independent train or external validation is described. Highly complex hybrid quantum-dilated-CNN/deep neuro-fuzzy architecture relative to the very small patient-level sample.
Wang et al., 2022 [40]	Low	Low	Low	High	High	Well-matched case–control design with histologically confirmed neoplasm; however, only 43 neoplasm cases, threefold cross-validation only, no independent external test set.
Wang, Chen, Lee & Fang, 2024 [41]	Low	Low	Low	High	High	Voice signal alone was explicitly insufficient (AUC 0.631 primary/0.470 external), with acceptable performance being reached only after adding demographic and structured clinical data, indicating substantial reliance on non-acoustic predictors rather than the voice signal itself.
Kim, Song, Park & Lee, 2024 [42]	High	Low	Low	High	High	Male-only, single-center cohort with a small cancer group versus much larger comparator groups; significant age and smoking-history imbalance between groups not adjusted for in modeling; audio segmented into fine-grained 0.5 s intervals before 5-fold cross-validation with no explicit statement that splitting was performed at the patientlevel, raising data-leakage risk; multiclass accuracy markedly lower than binary tasks, indicating substantial class overlap.
Kim, Dobko, Li et al., 2025 [44]	High	Unclear	Unclear	High	High	Malignant SCC cases pooled with benign polyps and cysts under a single “VF lesion” label, precluding cancer-specific performance assessment; small, imbalanced class sizes; two institutions used different microphones/hardware without controlling mouth-to-microphone distance; despite genuine external validation, multiclass accuracy collapsed to 35–45% on the external cohort.
Verikas et al., 2010 [45]	High	Low	Low	High	High	Cancerous class very small; genetic-search feature selection and SVM hyperparameter tuning optimized jointly and repeatedly on the same resampled data with no independent external test set, substantial optimism risk.
Mahmood, 2025 [46]	High	Low	Unclear	High	High	Single database (SVD) only, no external validation; heavy synthetic data augmentation (500 samples/class) with unclear separation from original recordings
Kwon et al., 2025 [47]	High	Low	Low	High	High	Male-only, age->40 restricted cohort; external validation n = 64 (30 cancer) used to both boost/train the decision-tree ensemble and evaluate it, same small sample serves dual purpose, inflating apparent accuracy; no separate held-out test set.
Sahoo, Mishra, Brahma & Bhoi, 2025 [48]	High	Low	High	High	High	no histological or clinical confirmation of malignancy is reported. SVD and MEEI cohorts pooled despite differing recording protocols. Small, unevenly distributed samples, only threefold cross-validation, no independent external test cohort.
Reid et al., 2022 [49]	High	Low	Low	High	High	Tiny external clinical validation (n = 30); recording protocol differs materially between SVD training data and external clinic data, domain-shift risk; wide, unstable 95% CIs on external validation metrics.
Kanagachalam & Kim, 2025 [50]	High	Low	High	High	High	“Organic-structural (malignant)” class combines vocal cord carcinoma, leukoplakia and unspecified “tumors” without histological differentiation; the malignant class was used only during meta-training and never independently evaluated in meta-testing, so reported accuracies do not reflect malignant-lesion detection performance; no independent patient-level external validation cohort.
Alonso et al., 2001 [51]	High	Low	Unclear	High	High	Pathological group is a heterogeneous mix of laryngeal diseases (nodules, polyps, carcinoma, paralysis, etc.); the seven new HOS-based parameters were selected using probability-density comparisons on the same dataset later used to report classifier accuracy, high risk of optimistic bias.
You et al., 2025 [52]	High	Low	Unclear	High	High	Severe class imbalance; test set contains only ~14 tumor cases; tumor-confirmation method (histology vs. clinical impression) not explicitly stated.

Note: ROB = Risk of Bias; SCC = squamous cell carcinoma; DNN = deep neural network; CNN = convolutional neural network; ML = machine learning; SVD = Saarbrücken Voice Database. “Unclear” indicates the study report did not provide sufficient information to permit a risk-of-bias judgment for that domain.

**Table 4:** Acoustic features analyzed across included studies.

Feature Category	Specific Features	Studies Used
Cepstral coefficients	MFCCs, delta MFCCs, LPCCs, BFCCs	Wang et al., 2022 [40]; Gour et al., 2020 [12]; You et al., 2025 [52]; Godino-Llorente et al., 2004 [17]; Kim et al., 2024 [42]; Sahoo et al., 2025 [48], Kim et al., 2025 [44]; BenAicha et al., 2024 [20]; Sachane et al., 2025 [39]; Song et al., 2024 [18]
Frequency measures	F0, formant frequencies (F1-F4), formant dispersion	Liu et al., 2025 [43]; Kim et al., 2025 [44]; Ben Aicha et al., 2024 [20]
Perturbation measures	Jitter, shimmer, APQ, PPQ	Godino-Llorente et al., 2004 [17]; Kim et al., 2025 [44]; Ben Aicha et al., 2024 [20]; Verikas et al., 2010 [45]
Noise measures	HNR, NNE, CPP	Godino-Llorente et al., 2004 [17]; Liu et al., 2025 [43]; Verikas et al., 2010 [45]; Ben Aicha et al., 2024 [20]
Spectral features	Spectral centroid, spectral spread, spectral skewness, RMSE	Liu et al., 2025 [43]; Sachane et al., 2025 [39]
Time-frequency representations	Mel-spectrograms, Gammatonegram, TKEO-Scalogram, CGT-Scalogram	Reid et al., 2021 [49]; Kanagachalam et al., 2025 [50]; Mahmood et al., 2025 [46]
Nonlinear features	Mutual information, correlation dimension, Renyi entropy	Gour et al., 2020 [12]
Higher-order statistics	Bicoherence indices, kurtosis interference	Alonso et al., 2001 [51]
Novel filter approaches	Octave Frequency Spectrum Energy (OFSE)	Song et al., 2024 [18]

Note: MFCCs = mel-frequency cepstral coefficients; LPCCs = linear predictive cepstral coefficients; BFCCs = bark frequency cepstral coefficients; F0 = fundamental frequency; APQ = amplitude perturbation quotient; PPQ = pitch perturbation quotient; HNR = harmonics-to-noise ratio; NNE = normalized noise energy; CPP = cepstral peak prominence; RMSE = root mean square error; TKEO = Teager–Kaiser energy operator; CGT = combined Gammatonegram.

**Table 5:** Performance metrics for binary pathological vs. normal voice classification.

Study	Method	Accuracy (%)	Sensitivity (%)	Specificity (%)	AUC	F1-Score (%)	Additional Metrics
Godino-Llorente & Gómez-Vilda, 2004 [17]	LVQ neural network	96.0	NR	NR	NR	NR	Frame accuracy; no misclassified records
Reid et al., 2021 [49]	VGG19 CNN + SVM	97.8	98.5	97.1	NR	NR	Clinical validation: 96.7%
Sahoo et al., 2025 [48]	IML-CNN	97.36	95.36	NR	NR	96.25	Recall reported
Mahmood et al., 2025 [46]	ResNet with CGT-Scalogram	96.0	96.1	NR	NR	NR	Precision: 96.3%
Alonso et al., 2001 [51]	Neural network with HOS	98.3	NR	NR	NR	NR	Combined classic and HOS parameters

Note: LVQ = learning vector quantization; CNN = convolutional neural network; SVM = support vector machine; IML-CNN = iterative multi-level convolutional neural network; CGT = combined Gammatonegram and TKEO; HOS = higher-order statistics; AUC = area under the curve; NR = not reported.

**Table 6:** Performance metrics for binary malignant vs. benign classification.

Study	Method	Modality	Accuracy (%)	Sensitivity (%)	Specificity (%)	AUC	Validation Method	Notes
Song et al., 2024 [18]	CNN with OFSE	Voice only	100.0	NR	NR	0.99	Internal validation	Healthy vs. cancer only
Wang et al., 2022 [40]	DNN with MFCC	Voice only	86.11	77.78	88.89	0.91	3-fold CV	Glottic neoplasm vs. benign
Wang et al., 2024 [41]	CNN-GRU-Attention	Voice + demographics + medical records	81.5	78.3	81.6	0.878	Internal validation	Multimodal integration
Wang et al., 2024 [41]	Voice analysis	Voice only	NR	NR	NR	0.631	Internal validation	Voice alone insufficient
Wang et al., 2024 [41]	Voice + demographics	Voice + demographics	NR	NR	NR	0.807	Internal validation	Improved over voice alone
Kwon et al., 2025 [47]	Ensemble decision tree	Image + voice	95.31	97.06	93.33	NR	Internal validation	Combined modalities
Kwon et al., 2025 [47]	CNN	Voice only	87.88	NR	NR	NR	Internal validation	Single modality
Kwon et al., 2025 [47]	CNN	Image only	89.06	NR	NR	NR	Internal validation	Single modality

Note: DNN = deep neural network; MFCC = mel-frequency cepstral coefficients; CNN = convolutional neural network; GRU = gated recurrent unit; OFSE = octave frequency spectrum energy; CV = cross-validation; AUC = area under the curve; NR = not reported.

**Table 7:** Performance metrics for multi-class pathology classification.

Study	Classes	Method	Accuracy (%)	Sensitivity/Recall (%)	Specificity (%)	Precision (%)	Binary Comparison
Kim et al., 2024 [42]	4-class (healthy, cancer, paralysis, benign)	Various ML algorithms	75–83	NR	NR	NR	Binary: 85–97%
You et al., 2025 [52]	2-class (tumor vs. non-tumor)	Siamese network	94.37	78.57	98.25	NR	N/A
You et al., 2025 [52]	3-class (normal, benign, tumor)	Siamese network	75.0	NR	NR	NR	25.8% drop from binary
Kim et al., 2025 [44]	3-class (HVFs, UVFP, VF lesions)	Deep learning	40.0	NR	NR	NR	Binary: 83%
Mahmood et al., 2025 [46]	Multi-class pathologies	ResNet with CGT	94.4	94.0	NR	94.5	N/A
Verikas et al., 2010 [45]	3-class (healthy, cancerous, noncancerous)	Multiple classifiers	>92.0	NR	NR	NR	Questionnaire data superior
Gour et al., 2020 [12]	2-class (cancer vs. healthy)	Random forest	80.0	NR	NR	NR	N/A
Kanagachalam & Kim, 2025 [50]	6-class pathologies	Few-shot learning	NR	NR	NR	NR	Not explicitly reported

Note: ML = machine learning; HVFs = healthy vocal folds; UVFP = unilateral vocal fold paralysis; VF = vocal fold; CGT = combined Gammatonegram and TKEO; NR = not reported; N/A = not applicable.

**Table 8:** Machine learning and deep learning approaches utilized across studies.

Approach Category	Specific Methods	No. of Studies	Studies
Traditional Machine Learning	LVQ neural networks	1	Godino-Llorente & Gómez-Vilda, 2004 [17]
Traditional Machine Learning	Support vector machines (SVMs)	2	Reid et al., 2021 [51]; Verikas et al., 2010 [45]
Traditional Machine Learning	Ensemble decision trees	1	Kwon et al., 2025 [47]
Traditional Machine Learning	Random forest classifiers	1	Gour et al., 2020 [12]
Traditional Machine Learning	Neural networks with HOS	1	Alonso et al., 2001 [51]
Deep Learning-CNN	Standard CNN architectures	3	Song et al., 2024 [18]; Sachane & Patil, 2025 [39]; Kwon et al., 2025 [47]
Deep Learning-CNN	Transfer learning (VGG19)	1	Reid et al., 2021 [49]
Deep Learning-CNN	ResNet variants	1	Mahmood et al., 2025 [46]
Deep Learning-CNN	Iterative multi-level CNN	1	Sahoo et al., 2025 [48]
Deep Learning-Hybrid	CNN-GRU-Attention	1	Wang et al., 2024 [41]
Deep Learning-DNN	Deep neural networks	2	Wang et al., 2022 [40]; Kim et al., 2024 [42]
Deep Learning-Advanced	Siamese networks	1	You et al., 2025 [52]
Deep Learning-Advanced	Few-shot learning	1	Kanagachalam & Kim, 2025 [50]

Note: LVQ = learning vector quantization; HOS = higher-order statistics; CNN = convolutional neural network; DNN = deep neural network; GRU = gated recurrent unit.

**Table 9:** Comparative performance: traditional ML vs. deep learning for binary pathological-normal classification.

Approach Type	Study	Method	Accuracy (%)	Sensitivity (%)	Specificity (%)
Traditional ML	Godino-Llorente & Gómez-Vilda, 2004 [17]	LVQ neural network	96.0	NR	NR
Traditional ML	Alonso et al., 2001 [51]	Neural network + HOS	98.3	NR	NR
Deep Learning	Reid et al., 2021 [49]	VGG19 CNN + SVM	97.8	98.5	97.1
Deep Learning	Sahoo et al., 2025 [48]	IML-CNN	97.36	95.36	NR
Deep Learning	Mahmood et al., 2025 [46]	ResNet + CGT	96.0	96.1	NR

Note: ML = machine learning; LVQ = learning vector quantization; HOS = higher-order statistics; CNN = convolutional neural network; SVM = support vector machine; IML-CNN = iterative multi-level CNN; CGT = combined Gammatonegram and TKEO; NR = not reported.

**Table 10:** Performance metrics by specific algorithm architecture.

Architecture Type	Study	Specific Implementation	Classification Task	Accuracy (%)	AUC	Key Innovation
DNN	Wang et al., 2022 [40]	DNN + MFCC	Malignant vs. benign	86.11	0.91	Feature extraction optimization
CNN-RNN Hybrid	Wang et al., 2024 [41]	CNN-GRU-Attention	Malignant vs. benign (multimodal)	81.5	0.878	Multimodal integration
Transfer Learning	Reid et al., 2021 [49]	VGG19 (pretrained) + SVM	Pathological vs. normal	97.8	NR	Transfer from ImageNet
CNN Advanced	Sahoo et al., 2025 [48]	Iterative multi-level CNN	Pathological vs. normal	97.36	NR	Progressive feature refinement
CNN Advanced	Mahmood et al., 2025 [46]	ResNet + CGT-Scalogram	Pathological vs. normal	96.0	NR	Time–frequency fusion
CNN Novel Features	Song et al., 2024 [18]	CNN + OFSE	Healthy vs. cancer	100.0	0.99	Frequency-band optimization
Siamese Network	You et al., 2025 [52]	Siamese + contrastive learning	Tumor vs. non-tumor	94.37	NR	Small sample optimization
Ensemble	Kwon et al., 2025 [47]	Ensemble decision trees	Cancer detection (multimodal)	95.31	NR	Image + voice fusion

Note: DNN = deep neural network; MFCCs = mel-frequency cepstral coefficients; CNN = convolutional neural network; RNN = recurrent neural network; GRU = gated recurrent unit; SVM = support vector machine; CGT = combined Gammatonegram and TKEO; OFSE = octave frequency spectrum energy; AUC = area under the curve; NR = not reported.

## Data Availability

The original contributions presented in this study are included in the article/Supplementary Materials. Further inquiries can be directed to the corresponding authors.

## Supplementary Materials

The following supporting information can be downloaded at: https://www.mdpi.com/article/10.3390/medsci14050581/s1, File S1: Search Strategies; File S2: Data Extraction Sheet; File S3: Summary of the PRISMA 2020 Checklist Items; File S4: PROBAST Assessment.

## Abbreviations

ML = Machine Learning; SCC: = Squamous Cell Carcinoma; CIS = Carcinoma in situ; VF = Vocal Folds; VFL = Vocal Fold Lesion; UVFP = Unilateral vocal fold paralysis; ROB = Risk of Bias; DNN = Deep Neural Network; CNN = Convolutional neural network; SVD = Saarbrücken Voice Database; MFCC = mel-frequency cepstral coefficients; LPCCs = linear predictive cepstral coefficients; BFCC = bark frequency cepstral coefficients; APQ = amplitude perturbation quotient; PPQ = pitch perturbation quotient; HNR = harmonics-to-noise ratio; NNE = normalized noise energy; CPP = cepstral peak prominence; RMSE = root mean square error; TKEO = Teager–Kaiser energy operator; CGT = combined Gammatonegram; LVQ = learning vector quantization; SVM = support vector machine; IML-CNN = iterative multi-level convolutional neural network; HOS = higher-order statistics; AUC = area under the curve; NR = not reported.
