# Supplementary material for: Early Detection of Glottic Cancer Through Machine Learning and Acoustic Voice Analysis: A Systematic Review
Source: Med Sci (Basel). 2026 Sep 17;14(5):581. doi: 10.3390/medsci14050581 (PMC13609340; doi:10.3390/medsci14050581)
Supplement: Supplementary file 1 [file medsci-14-00581-s001.zip › Supplementary File S4.pdf]

Supplementary File S4: PROBAST Assessment - Detailed Evaluation of Included Studies

Assessment Tool: Prediction model Risk Of Bias ASsessment Tool (PROBAST)

Total Studies Assessed: 18

Reviewers: HV and RM (Independent assessment); BN (Adjudication)

Overall Risk of Bias Summary

Risk of Bias Category	Number of Studies	Percentage	Studies
Low Risk	1	5.6%	Kwon et al., 2025
Moderate Risk	13	72.2%	Wang et al. (2022, 2024), Liu et al. (2025), You et al. (2025), Kim et al. (2024), Sahoo et al. (2025), Ben Aicha et al. (2023), Alonso et al. (2001), Verikas et al. (2010), Reid et al. (2022), Sachane et al. (2024), Mahmood et al. (2025), Song et al. (2024)
High Risk	4	22.2%	Gour et al. (2020), Godino-Llorente et al. (2004), Kim et al. (2025), Kanagachalam et al. (2025)

Study 1: Wang et al., 2022

Study Type: Retrospective case-control

Population: Glottic neoplasm vs benign voice disorders

Overall Risk of Bias: MODERATE

Domain 1: Participants

Assessment Aspect	Evaluation	Notes
Risk of Bias	Moderate	43 malignancy cases; age and sex-matched design may not be robust for younger and female patients
Concerns about Applicability	Low	Population appropriate for review question

Domain 2: Predictors

Assessment Aspect	Evaluation	Notes
Risk of Bias	Low	MFCC features extracted consistently; standardized voice recording protocol
Concerns about Applicability	Low	Predictors match review question

Domain 3: Outcome

Assessment Aspect	Evaluation	Notes
Risk of Bias	Low	Histopathologically confirmed malignancy; clear outcome categories
Concerns about Applicability	Low	Outcome definition appropriate

Domain 4: Analysis

Assessment Aspect	Evaluation	Notes
Risk of Bias	Moderate	3-fold cross-validation; no external validation; sample size adequate but limited
Concerns about Applicability	Moderate	Internal validation only limits generalizability

---

Study 2: Liu et al., 2025

Study Type: Cross-sectional

Population: Normal, benign, malignant

Overall Risk of Bias: MODERATE

Domain 1: Participants

| Assessment Aspect | Evaluation | Notes |

Risk of Bias	Low	41 glottic carcinoma, 64 benign, 52 controls; adequate sample distribution
Concerns about Applicability	Low	Representative population

Domain 2: Predictors

Assessment Aspect	Evaluation	Notes
Risk of Bias	Low	Frequency measures, noise measures, spectral features; standardized extraction
Concerns about Applicability	Low	Appropriate predictor selection

Domain 3: Outcome

Assessment Aspect	Evaluation	Notes
Risk of Bias	Low	Clear diagnostic categories with histological confirmation
Concerns about Applicability	Low	Outcome matches review question

Domain 4: Analysis

Assessment Aspect	Evaluation	Notes
Risk of Bias	Moderate	PCA approach; internal validation only; no external dataset testing
Concerns about Applicability	Moderate	Lack of external validation

---

Study 3: Gour et al., 2020

Study Type: Case-control

Population: Laryngeal cancer vs healthy

Overall Risk of Bias: HIGH

Domain 1: Participants

Assessment Aspect	Evaluation	Notes
Risk of Bias	Moderate	55 cancer cases vs 55 controls; male-only population limits generalizability
Concerns about Applicability	Low	Appropriate case selection despite gender limitation

Domain 2: Predictors

Assessment Aspect	Evaluation	Notes
Risk of Bias	Low	MFCC and nonlinear features (mutual information, correlation dimension, Renyi entropy)
Concerns about Applicability	Low	Appropriate acoustic features

Domain 3: Outcome

Assessment Aspect	Evaluation	Notes
Risk of Bias	Low	Confirmed laryngeal cancer diagnoses
Concerns about Applicability	Low	Clear outcome definition

Domain 4: Analysis

Assessment Aspect	Evaluation	Notes
Risk of Bias	High	Random forest classifier; no external validation; limited sample size; 80% accuracy suggests overfitting risk
Concerns about Applicability	High	High risk of overfitting with no external validation

---

Study 4: You et al., 2025

Study Type: Case-control

Population: Tumor vs non-tumor

Overall Risk of Bias: MODERATE

Domain 1: Participants

Assessment Aspect	Evaluation	Notes
Risk of Bias	Low	71 tumor cases; includes nodules, polyps, cysts in non-tumor group
Concerns about Applicability	Low	Appropriate participant selection

Domain 2: Predictors

Assessment Aspect	Evaluation	Notes
Risk of Bias	Moderate	Recording environment not fully specified; feature extraction using Siamese network
Concerns about Applicability	Low	Predictors appropriate despite incomplete documentation

Domain 3: Outcome

Assessment Aspect	Evaluation	Notes
Risk of Bias	Low	Clear tumor vs non-tumor classification
Concerns about Applicability	Low	Outcome definition clear

Domain 4: Analysis

Assessment Aspect	Evaluation	Notes
Risk of Bias	Moderate	Siamese network with contrastive learning; internal validation; 25.8% accuracy drop in 3-class vs 2-class
Concerns about Applicability	Moderate	Performance degradation in multi-class suggests model limitations

---

Study 5: Wang et al., 2024

Study Type: Cross-sectional

Population: Glottic neoplasm vs benign disorders

Overall Risk of Bias: MODERATE

Domain 1: Participants

Assessment Aspect	Evaluation	Notes
Risk of Bias	Low	60 malignancy (SCC, CIS, dysplasia), 1940 benign; large benign cohort
Concerns about Applicability	Low	Excellent sample size and distribution

Domain 2: Predictors

Assessment Aspect	Evaluation	Notes
Risk of Bias	Low	Multimodal: voice + demographics + medical records; standardized protocols
Concerns about Applicability	Low	Comprehensive predictor set

Domain 3: Outcome

Assessment Aspect	Evaluation	Notes
Risk of Bias	Low	Histopathologically confirmed neoplasm; clear categories
Concerns about Applicability	Low	Gold standard outcome definition

Domain 4: Analysis

| Assessment Aspect | Evaluation | Notes |

|------------------|------------|-------|
| Risk of Bias | Moderate | CNN-GRU-Attention architecture; internal validation; demonstrated voice-only AUC 0.631 vs multimodal 0.878 |
| Concerns about Applicability | Low | Strong methodology but lacks external validation |

Study 6: Godino-Llorente et al., 2004

Study Type: Cross-sectional

Population: Pathological vs normal voices

Overall Risk of Bias: HIGH

Domain 1: Participants

Assessment Aspect	Evaluation	Notes
Risk of Bias	High	Only 3 carcinoma cases; extremely small malignancy cohort limits reliability
Concerns about Applicability	High	Insufficient cancer cases for robust conclusions

Domain 2: Predictors

Assessment Aspect	Evaluation	Notes
Risk of Bias	Moderate	Cepstral coefficients, perturbation measures, noise measures; used SVD database
Concerns about Applicability	Low	Appropriate features despite database concerns

Domain 3: Outcome

Assessment Aspect	Evaluation	Notes
Risk of Bias	Low	Clear pathological vs normal categories

| Concerns about Applicability | Low | Outcome definition appropriate |

Domain 4: Analysis

Assessment Aspect	Evaluation	Notes
Risk of Bias	High	LVQ neural network; 96% accuracy with only 3 cancer cases raises serious overfitting concerns; frame-based analysis
Concerns about Applicability	High	Results not generalizable due to extreme sample size limitation

---

Study 7: Kim et al., 2024

Study Type: Retrospective

Population: Laryngeal cancer, paralysis, benign, healthy

Overall Risk of Bias: MODERATE

Domain 1: Participants

Assessment Aspect	Evaluation	Notes
Risk of Bias	Low	30 early-stage SCC, 81 benign, 155 controls; adequate distribution
Concerns about Applicability	Low	Representative sample

Domain 2: Predictors

Assessment Aspect	Evaluation	Notes
Risk of Bias	Low	MFCC features; standardized voice recording protocols
Concerns about Applicability	Low	Standard feature extraction

Domain 3: Outcome

Assessment Aspect	Evaluation	Notes
Risk of Bias	Low	Histologically confirmed SCC; clear outcome categories
Concerns about Applicability	Low	Gold standard diagnosis

Domain 4: Analysis

Assessment Aspect	Evaluation	Notes
Risk of Bias	Moderate	DNN architecture; 4-class accuracy 75-83% vs binary 85-97%; internal validation only
Concerns about Applicability	Moderate	Multi-class performance degradation; no external validation

---

Study 8: Sahoo et al., 2025

Study Type: Retrospective

Population: Pathological vs normal

Overall Risk of Bias: MODERATE

Domain 1: Participants

Assessment Aspect	Evaluation	Notes
Risk of Bias	Moderate	Malignancy cases not specified separately from total pathological group
Concerns about Applicability	Moderate	Lack of specific malignancy reporting

Domain 2: Predictors

Assessment Aspect	Evaluation	Notes

Risk of Bias	Low	MFCC features; iterative multi-level CNN approach
Concerns about Applicability	Low	Appropriate predictor methodology

Domain 3: Outcome

Assessment Aspect	Evaluation	Notes
Risk of Bias	Moderate	Pathological vs normal; lack of specific malignancy subgroup reporting
Concerns about Applicability	Low	General pathology classification appropriate

Domain 4: Analysis

Assessment Aspect	Evaluation	Notes
Risk of Bias	Moderate	IML-CNN achieving 97.36% accuracy; internal validation; no external testing
Concerns about Applicability	Moderate	High accuracy but limited validation

---

Study 9: Kim et al., 2025

Study Type: Retrospective

Population: UVFP, VF lesions, healthy

Overall Risk of Bias: HIGH

Domain 1: Participants

Assessment Aspect	Evaluation	Notes
Risk of Bias	Moderate	Only 14 SCC cases; small malignancy cohort
Concerns about Applicability	Low	Population matches question despite small size

Domain 2: Predictors

Assessment Aspect	Evaluation	Notes
Risk of Bias	Low	Formant parameters, MFCCs; Kernel SHAP analysis for feature importance
Concerns about Applicability	Low	Comprehensive feature analysis

Domain 3: Outcome

Assessment Aspect	Evaluation	Notes
Risk of Bias	Low	Confirmed SCC diagnosis; clear categories
Concerns about Applicability	Low	Appropriate outcome definition

Domain 4: Analysis

Assessment Aspect	Evaluation	Notes
Risk of Bias	High	Deep learning; 3-class accuracy only 40% vs binary 83%; small cancer cohort with complex model
Concerns about Applicability	High	Severe overfitting risk; poor multi-class performance

---

Study 10: Ben Aicha et al., 2023

Study Type: Cross-sectional

Population: Premalignant lesions vs normal

Overall Risk of Bias: MODERATE

Domain 1: Participants

Assessment Aspect	Evaluation	Notes

Risk of Bias	Low	93 premalignant (leukoplakia, erythroplakia, keratosis), 54 controls
Concerns about Applicability	Low	Excellent sample size for premalignant lesions

Domain 2: Predictors

Assessment Aspect	Evaluation	Notes
Risk of Bias	Low	F0, formants, perturbation measures, noise measures, MFCCs; comprehensive feature set
Concerns about Applicability	Low	Thorough acoustic analysis

Domain 3: Outcome

Assessment Aspect	Evaluation	Notes
Risk of Bias	Low	Premalignant lesions vs normal; histologically confirmed
Concerns about Applicability	Low	Clear outcome definition

Domain 4: Analysis

Assessment Aspect	Evaluation	Notes
Risk of Bias	Moderate	94% accuracy, 95% recall, 89% precision; internal validation; mutual information analysis
Concerns about Applicability	Moderate	Good performance but no external validation

---

Study 11: Alonso et al., 2001

Study Type: Case-control

Population: Pathological vs healthy

Overall Risk of Bias: MODERATE

Domain 1: Participants

Assessment Aspect	Evaluation	Notes
Risk of Bias	Moderate	Carcinoma cases included but exact number not specified; 68 benign, 100 controls
Concerns about Applicability	Moderate	Lack of specific case numbers

Domain 2: Predictors

Assessment Aspect	Evaluation	Notes
Risk of Bias	Low	Classic acoustic parameters + higher-order statistics (bicoherence, kurtosis)
Concerns about Applicability	Low	Novel feature approach appropriate

Domain 3: Outcome

Assessment Aspect	Evaluation	Notes
Risk of Bias	Low	Pathological vs healthy categories
Concerns about Applicability	Low	Clear outcome definition

Domain 4: Analysis

Assessment Aspect	Evaluation	Notes
Risk of Bias	Moderate	Neural network; 98.3% accuracy; no external validation; older methodology (2001)
Concerns about Applicability	Moderate	Dated methodology; internal validation only

---

Study 12: Kwon et al., 2025

Study Type: Cross-sectional

Population: Cancer vs non-cancer

Overall Risk of Bias: LOW

Domain 1: Participants

Assessment Aspect	Evaluation	Notes
Risk of Bias	Low	62 SCC; polyps (71), nodules (32), cysts; adequate sample sizes
Concerns about Applicability	Low	Excellent sample distribution

Domain 2: Predictors

Assessment Aspect	Evaluation	Notes
Risk of Bias	Low	Multimodal: laryngeal images + voice; standardized protocols
Concerns about Applicability	Low	Comprehensive multimodal approach

Domain 3: Outcome

Assessment Aspect	Evaluation	Notes
Risk of Bias	Low	Histologically confirmed SCC
Concerns about Applicability	Low	Gold standard diagnosis

Domain 4: Analysis

Assessment Aspect	Evaluation	Notes
Risk of Bias	Low	Ensemble decision trees; 95.31% accuracy with multimodal; demonstrated superiority over unimodal (voice 87.88%, image 89.06%)
Concerns about Applicability	Low	Robust methodology with clear multimodal benefit

---

Study 13: Verikas et al., 2010

Study Type: Cross-sectional

Population: Cancerous, non-cancerous, healthy

Overall Risk of Bias: MODERATE

Domain 1: Participants

Assessment Aspect	Evaluation	Notes
Risk of Bias	Moderate	22 carcinoma cases (small); 193 benign, 25 controls
Concerns about Applicability	Low	Representative despite small cancer cohort

Domain 2: Predictors

Assessment Aspect	Evaluation	Notes
Risk of Bias	Low	Perturbation measures, noise measures; questionnaire data included
Concerns about Applicability	Low	Comprehensive predictor set

Domain 3: Outcome

Assessment Aspect	Evaluation	Notes
Risk of Bias	Low	Three-class pathology categories
Concerns about Applicability	Low	Clear outcome categories

Domain 4: Analysis

Assessment Aspect	Evaluation	Notes
Risk of Bias	Moderate	Multiple classifiers; >92% accuracy; found questionnaire data superior to voice features; small cancer cohort
Concerns about Applicability	Moderate	Limited cancer cases; reliance on questionnaire data

---

Study 14: Reid et al., 2022

Study Type: Cross-sectional

Population: Pathological vs healthy

Overall Risk of Bias: MODERATE

Domain 1: Participants

Assessment Aspect	Evaluation	Notes
Risk of Bias	Moderate	Sample sizes not specified separately for malignancy
Concerns about Applicability	Moderate	Lack of detailed sample reporting

Domain 2: Predictors

Assessment Aspect	Evaluation	Notes
Risk of Bias	Low	Mel-spectrograms; used Saarbrücken Voice Database (German speakers)
Concerns about Applicability	Moderate	Cross-linguistic applicability uncertain

Domain 3: Outcome

Assessment Aspect	Evaluation	Notes
Risk of Bias	Low	Pathological vs healthy
Concerns about Applicability	Low	Clear outcome definition

Domain 4: Analysis

Assessment Aspect	Evaluation	Notes

| Risk of Bias | Low | VGG19 CNN + SVM; 97.8% accuracy; clinical validation maintained 96.7%; external validation performed |

| Concerns about Applicability | Moderate | External validation performed; acknowledged cross-linguistic uncertainty |

Study 15: Sachane et al., 2024

Study Type: Computational

Population: Cancerous vs healthy

Overall Risk of Bias: MODERATE

Domain 1: Participants

Assessment Aspect	Evaluation	Notes
Risk of Bias	Moderate	1320 patches (not individual cases); 259 controls; unclear how patches relate to actual patients
Concerns about Applicability	Moderate	Patch-based analysis vs patient-based

Domain 2: Predictors

Assessment Aspect	Evaluation	Notes
Risk of Bias	Low	MFCCs, spectral features (centroid, spread, skewness)
Concerns about Applicability	Low	Standard feature extraction

Domain 3: Outcome

Assessment Aspect	Evaluation	Notes
Risk of Bias	Moderate	Cancerous vs healthy; patch-based analysis may not reflect patient-level diagnosis
Concerns about Applicability	Moderate	Uncertainty in clinical translation

Domain 4: Analysis

Assessment Aspect	Evaluation	Notes
Risk of Bias	Moderate	CNN architecture; patch-based rather than patient-based evaluation; unclear validation strategy
Concerns about Applicability	High	Patch-level metrics may overestimate patient-level performance

---

Study 16: Kanagachalam et al., 2025

Study Type: Retrospective

Population: Six-class pathology

Overall Risk of Bias: HIGH

Domain 1: Participants

Assessment Aspect	Evaluation	Notes
Risk of Bias	Moderate	46 malignancy cases (carcinoma, leukoplakia, tumors); 89 laryngitis/polyps; 90 controls
Concerns about Applicability	Low	Appropriate sample despite complexity

Domain 2: Predictors

Assessment Aspect	Evaluation	Notes
Risk of Bias	Low	Gammatonegram time-frequency representations
Concerns about Applicability	Low	Novel feature approach

Domain 3: Outcome

Assessment Aspect	Evaluation	Notes
Risk of Bias	Low	Six distinct pathology classes
Concerns about Applicability	Low	Clear category definitions

Domain 4: Analysis

Assessment Aspect	Evaluation	Notes
Risk of Bias	High	Few-shot learning; performance metrics not explicitly reported; complex 6-class problem with limited reporting
Concerns about Applicability	High	Insufficient performance reporting; overfitting risk

---

Study 17: Mahmood et al., 2025

Study Type: Cross-sectional

Population: Pathological vs healthy

Overall Risk of Bias: MODERATE

Domain 1: Participants

Assessment Aspect	Evaluation	Notes
Risk of Bias	Low	128 malignancy, 445 benign, 350 controls; large sample sizes
Concerns about Applicability	Low	Excellent sample distribution

Domain 2: Predictors

Assessment Aspect	Evaluation	Notes
Risk of Bias	Low	Combined Gammatonegram and TKEO-Scalogram

| Concerns about Applicability | Moderate | Used Saarbrücken Voice Database (SVD) - German speakers |

Domain 3: Outcome

Assessment Aspect	Evaluation	Notes
Risk of Bias	Low	Multi-class pathologies
Concerns about Applicability	Low	Clear outcome categories

Domain 4: Analysis

Assessment Aspect	Evaluation	Notes
Risk of Bias	Moderate	ResNet architecture; 94.4% multi-class accuracy; specific pathology categories not clearly delineated; internal validation
Concerns about Applicability	Moderate	Good performance but lacks external validation

---

Study 18: Song et al., 2024

Study Type: Cross-sectional

Population: Laryngeal cancer, benign, paralysis, healthy

Overall Risk of Bias: MODERATE

Domain 1: Participants

Assessment Aspect	Evaluation	Notes
Risk of Bias	Low	30 glottis/transglottis cancer, 81 benign, 155 controls
Concerns about Applicability	Low	Adequate sample distribution

Domain 2: Predictors

Assessment Aspect	Evaluation	Notes
Risk of Bias	Low	Novel OFSE (Octave Frequency Spectrum Energy) features; demonstrated superiority over traditional MFCCs
Concerns about Applicability	Low	Innovative feature approach with strong validation

Domain 3: Outcome

Assessment Aspect	Evaluation	Notes
Risk of Bias	Low	Multiple categories: cancer, benign mucosal disease, paralysis, healthy
Concerns about Applicability	Low	Comprehensive outcome categories

Domain 4: Analysis

Assessment Aspect	Evaluation	Notes
Risk of Bias	Moderate	CNN architecture; 100% accuracy for healthy vs cancer but limited to binary; internal validation; over-formant frequencies (>4kHz) most discriminative
Concerns about Applicability	Moderate	Excellent binary performance but no external validation

---

PROBAST Domain Definitions and Assessment Criteria

Domain 1: Participants

Risk of Bias Questions:

- Were appropriate data sources used (e.g., cohort, RCT, or nested case-control design)?

- Were all inclusions and exclusions of participants appropriate?

Applicability: Are there concerns that the included participants do not match the review question?

Key Considerations for Voice-Based Glottic Cancer Studies:

- Adequacy of the number of malignancy cases relative to outcome events
- Clarity and appropriateness of inclusion and exclusion criteria
- Risks of selection bias and quality of group matching
- Representativeness of the included population for the intended clinical context

---

Domain 2: Predictors

Risk of Bias Questions:

- Were predictors defined and assessed in a similar way for all participants?
- Were predictor assessments made without knowledge of outcome data?
- Are all predictors available at the time the model is intended to be used?

Applicability: Are there concerns that the definition, assessment, or timing of predictors in the model do not match the review question?

Key Considerations for Voice-Based Studies:

- Consistency and standardization of voice recording procedures (sound-treated vs routine clinic rooms, microphone type, distance, sampling rate, voice tasks)
- Uniformity and transparency of acoustic feature extraction procedures
- Whether predictor measurements were obtained without knowledge of outcomes
- Whether all features would be obtainable in real-world clinical use

---

Domain 3: Outcome

Risk of Bias Questions:

- Was the outcome determined appropriately?

- Was a pre-specified or standard outcome definition used?

- Were predictors excluded from the outcome definition?

- Was the outcome defined and determined in a similar way for all participants?

- Was the outcome determination made without knowledge of predictor information?

- Was the time interval between predictor assessment and outcome determination appropriate?

Applicability: Are there concerns that the outcome, its definition, timing, or determination do not match the review question?

Key Considerations for Glottic Cancer Studies:

- Appropriateness of the reference standard, with particular attention to histopathological confirmation of malignancy

- Pre-specification and clarity of outcome categories (malignant, benign, normal or premalignant)

- Independence of outcome adjudication from predictor measurement to avoid incorporation bias

---

Domain 4: Analysis

Risk of Bias Questions:

- Were there a reasonable number of participants with the outcome?

- Were continuous and categorical predictors handled appropriately?

- Were all enrolled participants included in the analysis?

- Were participants with missing data handled appropriately?

- Was selection of predictors based on univariable analysis avoided?

- Were complexities in the data (e.g., censoring, competing risks, sampling of controls) accounted for appropriately?

- Were relevant model performance measures evaluated appropriately?

- Were model overfitting and optimism in model performance accounted for?

- Do predictors and their assigned weights in the final model correspond to the results from the reported multivariable analysis?

Applicability: Are there concerns that the analysis does not match the review question?

Key Considerations for Machine Learning Models:

- Suitability of sample size in relation to model complexity (e.g., events-per-variable ratio for conventional ML, parameter counts in deep learning models)

- Validity of the validation strategy (internal cross-validation vs external validation using independent datasets)

- Handling of missing data and avoidance of data leakage between training and test sets

- Completeness of performance reporting (sensitivity, specificity, accuracy, AUC, confidence intervals, and calibration indices)

- For deep learning models, the balance between the number of trainable parameters and the volume of training data

---

Key Findings from PROBAST Assessment

Common Sources of Bias

1. Limited External Validation (14/18 studies = 77.8%)
   - Most studies relied exclusively on internal cross-validation
   - Only 4 studies performed formal external validation
   - This limits assessment of true generalization capabilities

2. Small Malignancy Cohorts
   - Several studies had very small cancer sample sizes (3-30 cases)
   - Raises concerns about overfitting and generalizability
   - Events-per-variable ratios often inadequate for complex models

3. Database Dependencies
   - Heavy reliance on Saarbrücken Voice Database (German speakers)
   - Concerns about cross-linguistic applicability
   - Limited diversity in recording environments and protocols

4. Incomplete Reporting
   - Variable documentation of recording protocols
   - Inconsistent reporting of sample characteristics
   - Missing performance metrics in some studies

Strengths Identified

1. Histopathological Confirmation
   - Most studies used gold standard outcome determination
   - Clear diagnostic categories in majority of studies

2. Standardized Feature Extraction
   - Many studies employed well-established acoustic features
   - Transparent predictor measurement procedures

3. Multimodal Approaches
   - Studies combining voice with other data showed improved performance
   - Demonstrates value of comprehensive assessment

Recommendations for Future Research

Based on this PROBAST assessment, future studies should:

1. Prioritize External Validation
   - Use independent, prospective datasets
   - Test across multiple institutions and populations
   - Validate across different languages and recording environments

2. Ensure Adequate Sample Sizes
   - Calculate required sample sizes based on model complexity
   - Maintain appropriate events-per-variable ratios
   - Include sufficient malignancy cases for robust training

3. Standardize and Report Protocols
   - Document all recording parameters comprehensively
   - Specify microphone specifications, distances, environments
   - Report all voice tasks and preprocessing steps

4. Address Cross-Linguistic Concerns
   - Include diverse language populations
   - Test model performance across linguistic groups
   - Investigate language-specific acoustic patterns

5. Implement Prospective Designs
   - Move beyond retrospective analyses
   - Integrate models into real clinical workflows
   - Measure clinical impact and outcomes

Conclusion

This PROBAST assessment reveals that while the included studies demonstrate methodological diversity and innovation in applying AutoML and machine learning to glottic

cancer detection, the evidence base is limited by predominance of moderate-to-high risk of bias studies (94.4%), primarily due to lack of external validation, small malignancy cohorts, and database dependencies. Only one study (Kwon et al., 2025) achieved low overall risk of bias through robust multimodal methodology and adequate sample sizes. Future research must prioritize external validation, larger and more diverse datasets, and standardized protocols to move these promising technologies toward clinical implementation.
