# Supplementary material for: Early Detection of Glottic Cancer Through Machine Learning and Acoustic Voice Analysis: A Systematic Review
Source: Med Sci (Basel). 2026 Sep 17;14(5):581. doi: 10.3390/medsci14050581 (PMC13609340; doi:10.3390/medsci14050581)
Supplement: Supplementary file 1 [file medsci-14-00581-s001.zip › Supplementary File S1.pdf]

Supplementary File S1: Search Strategies

PubMed

("Glottic Neoplasms"[MeSH] OR "Laryngeal Neoplasms"[MeSH] OR glottic cancer*[tiab] OR glottic carcinoma*[tiab] OR glottic tumour*[tiab] OR glottic tumor*[tiab] OR laryngeal cancer*[tiab] OR laryngeal carcinoma*[tiab] OR vocal cord cancer*[tiab] OR vocal fold cancer*[tiab] OR "cancer of the glottis"[tiab]) AND ("Machine Learning"[MeSH] OR machine learning[tiab] OR deep learning[tiab] OR neural network*[tiab] OR "Artificial Intelligence"[MeSH] OR artificial intelligence[tiab] OR automated learning[tiab] OR autoML[tiab] OR automated machine learning[tiab] OR supervised learning[tiab] OR unsupervised learning[tiab] OR random forest*[tiab] OR support vector machine*[tiab] OR SVM[tiab] OR convolutional neural network*[tiab] OR CNN[tiab] OR decision tree*[tiab] OR ensemble method*[tiab]) AND ("Voice"[MeSH] OR "Speech"[MeSH] OR "Acoustics"[MeSH] OR acoustic feature*[tiab] OR voice feature*[tiab] OR speech feature*[tiab] OR acoustic analysis[tiab] OR voice analysis[tiab] OR speech analysis[tiab] OR acoustic parameter*[tiab] OR voice parameter*[tiab] OR fundamental frequency[tiab] OR F0[tiab] OR pitch[tiab] OR jitter[tiab] OR shimmer[tiab] OR harmonics to noise ratio[tiab] OR HNR[tiab] OR formant*[tiab] OR spectral feature*[tiab] OR cepstral[tiab] OR MFCC[tiab] OR mel frequency[tiab] OR AVQI[tiab] OR Acoustic Voice Quality Index[tiab]) AND ("Early Detection of Cancer"[MeSH] OR early detection[tiab] OR early diagnosis[tiab] OR screening[tiab] OR early stage[tiab])

Scopus:

("glottic cancer*" OR "glottic carcinoma*" OR "glottic tumour*" OR "glottic tumor*" OR "laryngeal cancer*" OR "laryngeal carcinoma*" OR "vocal cord cancer*" OR "vocal fold cancer*" OR "cancer of the glottis") OR INDEXTERMS("Glottic Neoplasms" OR "Laryngeal Neoplasms") AND ("machine learning" OR "deep learning" OR "neural network*" OR "artificial intelligence" OR "automated learning" OR "autoML" OR "automated machine learning" OR "supervised learning" OR "unsupervised learning" OR "random forest*" OR "support vector machine*" OR "SVM" OR "convolutional neural network*" OR "CNN" OR "decision tree*" OR "ensemble method*") OR INDEXTERMS("Machine Learning" OR "Artificial Intelligence") AND ("acoustic feature*" OR "voice feature*" OR "speech feature*" OR "acoustic analysis" OR "voice analysis" OR "speech analysis" OR "acoustic parameter*" OR "voice parameter*" OR "fundamental frequency" OR "F0" OR "pitch" OR "jitter" OR "shimmer" OR "harmonics to noise ratio" OR "HNR" OR "formant*" OR "spectral feature*" OR "cepstral" OR "MFCC" OR "mel frequency" OR "AVQI" OR "Acoustic Voice Quality Index") OR INDEXTERMS("Voice" OR "Speech" OR "Acoustics") AND ("early detection" OR "early diagnosis" OR "screening" OR "early stage") OR INDEXTERMS("Early Detection of Cancer")

EBSCO:

(MH "Glottic Neoplasms" OR MH "Laryngeal Neoplasms" OR TI ("glottic cancer*" OR "glottic carcinoma*" OR "glottic tumour*" OR "glottic tumor*" OR "laryngeal cancer*" OR "laryngeal carcinoma*" OR "vocal cord cancer*" OR "vocal fold cancer*" OR "cancer of the glottis") OR AB ("glottic cancer*" OR "glottic carcinoma*" OR "glottic tumour*" OR "glottic tumor*" OR "laryngeal cancer*" OR "laryngeal carcinoma*" OR "vocal cord cancer*" OR "vocal fold cancer*" OR "cancer of the glottis")) AND (MH "Machine Learning" OR MH "Artificial Intelligence" OR TI ("machine learning" OR "deep learning" OR "neural network*" OR "artificial intelligence" OR "automated learning" OR "autoML" OR "automated machine learning" OR "supervised learning" OR "unsupervised learning" OR "random forest*" OR "support vector machine*" OR SVM OR "convolutional neural network*" OR CNN OR "decision tree*" OR "ensemble method*") OR AB ("machine learning" OR "deep learning" OR "neural network*" OR "artificial intelligence" OR "automated learning" OR "autoML" OR "automated machine learning" OR "supervised learning" OR "unsupervised learning" OR "random forest*" OR "support vector machine*" OR SVM OR "convolutional neural network*" OR CNN OR "decision tree*" OR "ensemble method*")) AND (MH "Voice" OR MH "Speech" OR MH "Acoustics" OR TI ("acoustic feature*" OR "voice feature*" OR "speech feature*" OR "acoustic analysis" OR "voice analysis" OR "speech analysis" OR "acoustic parameter*" OR "voice parameter*" OR "fundamental frequency" OR F0 OR pitch OR jitter OR shimmer OR "harmonics to noise ratio" OR HNR OR formant* OR "spectral feature*" OR cepstral OR MFCC OR "mel frequency" OR AVQI OR "Acoustic Voice Quality Index") OR AB ("acoustic feature*" OR "voice feature*" OR "speech feature*" OR "acoustic analysis" OR "voice analysis" OR "speech analysis" OR "acoustic parameter*" OR "voice parameter*" OR "fundamental frequency" OR F0 OR pitch OR jitter OR shimmer OR "harmonics to noise ratio" OR HNR OR formant* OR "spectral feature*" OR cepstral OR MFCC OR "mel frequency" OR AVQI OR "Acoustic Voice Quality Index")) AND (MH "Early Detection of Cancer" OR TI ("early detection" OR "early diagnosis" OR screening OR "early stage") OR AB ("early detection" OR "early diagnosis" OR screening OR "early stage"))

OVID:

1. exp Glottic Neoplasms/ or exp Laryngeal Neoplasms/ 2. (glottic cancer* or glottic carcinoma* or glottic tumour* or glottic tumor* or laryngeal cancer* or laryngeal carcinoma* or vocal cord cancer* or vocal fold cancer* or cancer of the glottis).ti,ab. 3. 1 or 2 4. exp Machine Learning/ or exp Artificial Intelligence/ 5. (machine learning or deep learning or neural network* or artificial intelligence or automated learning or autoML or automated machine learning or supervised learning or unsupervised learning or random forest* or support vector machine* or SVM or convolutional neural network* or CNN or decision tree* or ensemble method*).ti,ab. 6. 4 or 5 7. exp Voice/ or exp Speech/ or exp Acoustics/ 8. (acoustic feature* or voice feature* or speech feature* or acoustic analysis or voice analysis or speech analysis or acoustic parameter* or voice parameter* or fundamental frequency or F0 or pitch or jitter or shimmer or harmonics to noise ratio or HNR or formant* or spectral feature* or cepstral or MFCC or mel frequency or AVQI or Acoustic Voice Quality Index).ti,ab. 9. 7 or 8 10. exp Early Detection of Cancer/ 11. (early detection or early diagnosis or screening or early stage).ti,ab. 12. 10 or 11 13. 3 and 6 and 9 and 12
